# Isolated Hypoglossal Nerve Palsy Associated with Internal Carotid Artery Dissection: A Systematic Review

**DOI:** 10.3390/neurolint18080150

**Published:** 2026-08-14

**Authors:** Pasquale Frisina, Valeria Panebianco, Filippo Valentini, Antonio Iacuzio, Carlo Cavaliere, Umberto Romeo, Iacopo Carbone, Lucia Borghetti, Daniela Messineo

**Affiliations:** 1 Department of Radiological, Oncological and Pathological Sciences, Sapienza University of Rome, 00185 Rome, Italy; 2 Department of Sense Organs, Sapienza University of Rome, 00185 Rome, Italy; 3 Department of Life Sciences, Health and Healthcare Professions Link Campus University, 00165 Rome, Italy; 4 Department of Oral and Maxillofacial Sciences, Sapienza University of Rome, 00185 Rome, Italy

**Keywords:** internal carotid artery dissection, hypoglossal nerve palsy, cranial nerve XII, cranial neuropathy, tongue deviation, neurovascular imaging, differential diagnosis, head and neck disorders

## Abstract

**Background**: Isolated hypoglossal nerve palsy is a rare neurological condition with a broad differential diagnosis. Although neoplastic disorders are the most common cause, internal carotid artery dissection (ICAD) is an uncommon but clinically important and potentially reversible vascular etiology that is frequently underrecognized. This systematic review synthesized the available evidence regarding the clinical presentation, imaging findings, pathophysiological mechanisms, treatment, and outcomes of isolated hypoglossal nerve palsy associated with ICAD through a standardized patient-level descriptive analysis. **Methods**: This systematic review was conducted according to the PRISMA 2020 guidelines and prospectively registered in PROSPERO (CRD420261364863). PubMed, Scopus, Web of Science, and the Cochrane Library were searched from inception to April 2026. Manual reference screening and forward citation tracking were also performed. Studies reporting original, patient-level data on isolated hypoglossal nerve palsy associated with ICAD were eligible. Clinical, imaging, treatment, and outcome data were extracted and synthesized descriptively. In hybrid publications combining original case reporting with a narrative literature review, only eligible, original patient data were included in the quantitative synthesis, whereas the narrative review components were used for citation tracking and contextual comparison. **Results**: A total of 26 standalone primary publications were included, comprising 23 case reports and 3 case series. Two additional hybrid publications each contributed one eligible patient. One hybrid publication contained an original case-report component, whereas the other reported two original cases and was therefore considered to contain a case-series component, although only one of its patients fulfilled the eligibility criteria. Overall, the 28 publications contributed 32 eligible patients to the patient-level quantitative synthesis. The narrative review components of the two hybrid publications were used solely for citation tracking and contextual comparison. Patients were predominantly male (28/32, 87.5%), with a median age of 47 years. Tongue deviation (28/28, 100%) and tongue weakness (30/30, 100%) were the hallmark clinical features, followed by dysarthria (26/26, 100%) and dysphagia (18/22, 81.8%). All patients presented with isolated hypoglossal nerve palsy at presentation, without Horner syndrome or additional lower cranial nerve involvement. MRI was the most frequently reported imaging modality (29/32, 90.6%), whereas CTA was commonly used to characterize luminal abnormalities. Intramural hematoma (25/26, 96.2%), luminal stenosis (23/26, 88.5%), and external arterial enlargement (22/24, 91.7%) were the most consistent neuroradiological findings. Conservative medical management predominated (31/32, 96.9%). The available evidence was more consistent with a compressive mechanism related to subadventitial arterial wall expansion, although an ischaemic contribution involving the vasa nervorum could not be excluded. A favourable clinical outcome (complete recovery or marked improvement) was observed in 21/30 patients (70.0%). Overall, improvement of any degree, including partial recovery, was documented in 25/30 evaluable patients (83.3%). Outcome data should be interpreted cautiously because they derive predominantly from individual case reports. **Conclusions**: This systematic review provides an updated standardized synthesis of the available case-level evidence on isolated hypoglossal nerve palsy associated with ICAD. CTA and MRI/MRA provide complementary diagnostic information and may facilitate timely diagnosis when interpreted according to the clinical context. The available evidence identifies recurrent clinico-radiological patterns that may assist clinicians in recognizing this uncommon presentation while highlighting the need for prospective multicentre studies to better inform future diagnostic and therapeutic strategies.

## 1. Introduction

Isolated hypoglossal nerve palsy is a rare but clinically significant neurological condition with a broad differential diagnosis. Its causes include neoplastic, vascular, infectious, inflammatory, traumatic, and idiopathic disorders, often making diagnostic evaluation challenging and leading to delayed diagnosis. Among these, neoplastic lesions involving the skull base, hypoglossal canal, or adjacent structures are the most common, particularly in patients with progressive or chronic symptoms [1].

Although less frequent, vascular aetiologies are of particular clinical importance because they are potentially reversible and may lead to serious neurological complications if not promptly recognized. Internal carotid artery dissection (ICAD), with an estimated annual incidence of 2.5–3 cases per 100,000 individuals, is a well-recognized cause of ischemic stroke in young and middle-aged adults, and an important cause of cranial neuropathies [2,3]. Cranial nerve involvement occurs in approximately 10–12% of ICAD cases, with the hypoglossal nerve representing the most frequently affected lower cranial nerve, accounting for up to 76% of dissection-related lower cranial neuropathies. This predilection reflects the close anatomical relationship between the extracranial internal carotid artery and the hypoglossal nerve at the skull base. Proposed mechanisms of hypoglossal nerve dysfunction include direct mechanical compression resulting from outward expansion of the dissected arterial wall and ischemic injury of the vasa nervorum, although the relative contribution of these mechanisms remains uncertain.

Despite this recognized association, isolated hypoglossal nerve palsy remains an uncommon and frequently overlooked presentation of ICAD, particularly in the absence of ischemic stroke, Horner syndrome, or other lower cranial nerve involvement. Patients may present only with tongue deviation, dysarthria, dysphagia, or hemilingual atrophy, features that are easily misattributed to otolaryngological, neuromuscular, or neoplastic disorders, thereby delaying diagnosis [4,5,6]. Hypoglossal nerve palsy may also occur in alternative vascular, syndromic, or idiopathic settings, including carotid aneurysms, vertebral or combined arterial dissections, Horner syndrome, Collet–Sicard syndrome, and Villaret syndrome [7,8,9,10,11,12,13].

Neurovascular imaging is central to the diagnosis of ICAD and to the evaluation of its underlying pathophysiology. Magnetic resonance imaging (MRI), particularly fat-suppressed T1-weighted sequences, together with magnetic resonance angiography (MRA), enables direct visualization of intramural hematoma and vessel-wall abnormalities, whereas computed tomography angiography (CTA) rapidly depicts luminal stenosis, occlusion, intimal flap, double-lumen appearance, and pseudoaneurysm formation. MRI/MRA primarily characterize vessel-wall abnormalities, whereas CTA is particularly useful for evaluating luminal abnormalities.

Because isolated hypoglossal nerve palsy secondary to ICAD is rare, the available evidence is largely limited to individual case reports, small case series, and a few narrative reviews. Consequently, the clinical spectrum, neuroradiological features, pathophysiological mechanisms, and diagnostic role of the different imaging modalities remain incompletely characterized.

Unlike previous narrative reviews, the present systematic review provides an updated patient-level synthesis of isolated hypoglossal nerve palsy associated with internal carotid artery dissection (ICAD), integrating detailed clinical characteristics with comprehensive neuroradiological findings, including imaging modalities, MRI sequences, vascular abnormalities, proposed pathophysiological mechanisms, treatment, and outcomes. Specifically, we aimed to characterize the clinical and neuroradiological presentation of affected patients, evaluate the proposed pathophysiological mechanisms, summarize the imaging findings reported across MRI, MRA, CTA, ultrasound, and angiography, and identify recurrent clinico-radiological patterns that may facilitate earlier recognition of this uncommon but clinically important presentation.

## 2. Materials and Methods

This systematic review was conducted in accordance with the Preferred Reporting Items for Systematic Reviews and Meta-Analyses (PRISMA 2020) guidelines. The review protocol was prospectively registered in the PROSPERO database (CRD420261364863), and the completed PRISMA 2020 Checklist is provided as Supplementary File S1.

A comprehensive literature search was performed in PubMed, Scopus, Web of Science, and the Cochrane Library from database inception to April 2026 to identify reports of hypoglossal nerve palsy associated with internal carotid artery dissection (ICAD). Two reviewers independently screened titles, abstracts, and full-text articles for eligibility. Disagreements were resolved through discussion and consensus. No publication date restrictions were applied. Studies published in English and other languages were considered eligible, provided that sufficient clinical information was available for patient-level data extraction.

The search strategy combined the terms “internal carotid artery dissection”, “hypoglossal nerve palsy”, “hypoglossal nerve”, “cranial nerve XII”, and related synonyms. Manual screening of reference lists and forward citation tracking were additionally performed to identify further eligible publications. The complete search strategies are reported in Supplementary Table S1.

Studies were considered eligible if they reported patients with hypoglossal nerve palsy associated with internal carotid artery dissection confirmed clinically and/or radiologically. The inclusion criteria for publications were as follows: original patient-level data on hypoglossal nerve palsy associated with clinically and/or radiologically confirmed internal carotid artery dissection. Eligible primary study designs encompassed case reports, case series, and observational studies reporting original patient-level data. Publications reporting multiple original patients were classified as case series according to their original study design, even when only one of the reported patients fulfilled the eligibility criteria of the present review. In such cases, the patient-level quantitative synthesis exclusively incorporated data pertaining to the eligible patient. Narrative reviews were not considered to be primary studies. In instances where a publication combined original patient reporting with a narrative literature review, the original and narrative components were considered separately. Eligible original patient data were included in the quantitative synthesis, whereas the narrative review component was used for reference-list screening, citation tracking, and contextual comparison.

For the purposes of this review, isolated hypoglossal nerve palsy was defined as involvement of the twelfth cranial nerve in the absence of concomitant involvement of cranial nerves IX, X, or XI, Horner syndrome, Collet–Sicard syndrome, or Villaret syndrome at presentation. Patients with delayed lower cranial nerve involvement after the initial presentation remained eligible.

Studies were excluded if hypoglossal nerve palsy was unrelated to internal carotid artery dissection, if only vertebral artery dissection was reported, if alternative causes (including neoplastic, infectious, or inflammatory disorders) were identified, or if the available clinical or imaging information was insufficient to confirm the diagnosis.

The following variables were extracted for each eligible patient: age, sex, vascular risk factors, potential triggering events, laterality of the dissection, clinical presentation, associated neurological findings, imaging modalities and MRI sequences, neuroradiological findings, time from symptom onset to diagnosis, initial specialty referral or misdiagnosis (when reported), treatment strategy and duration, clinical outcome, imaging follow-up, recurrence, and follow-up duration. Whenever necessary, the original reports were re-examined to verify patient-level data and resolve discrepancies identified during data extraction. The complete extracted dataset is provided in Supplementary Table S2.

**Figure 1:**
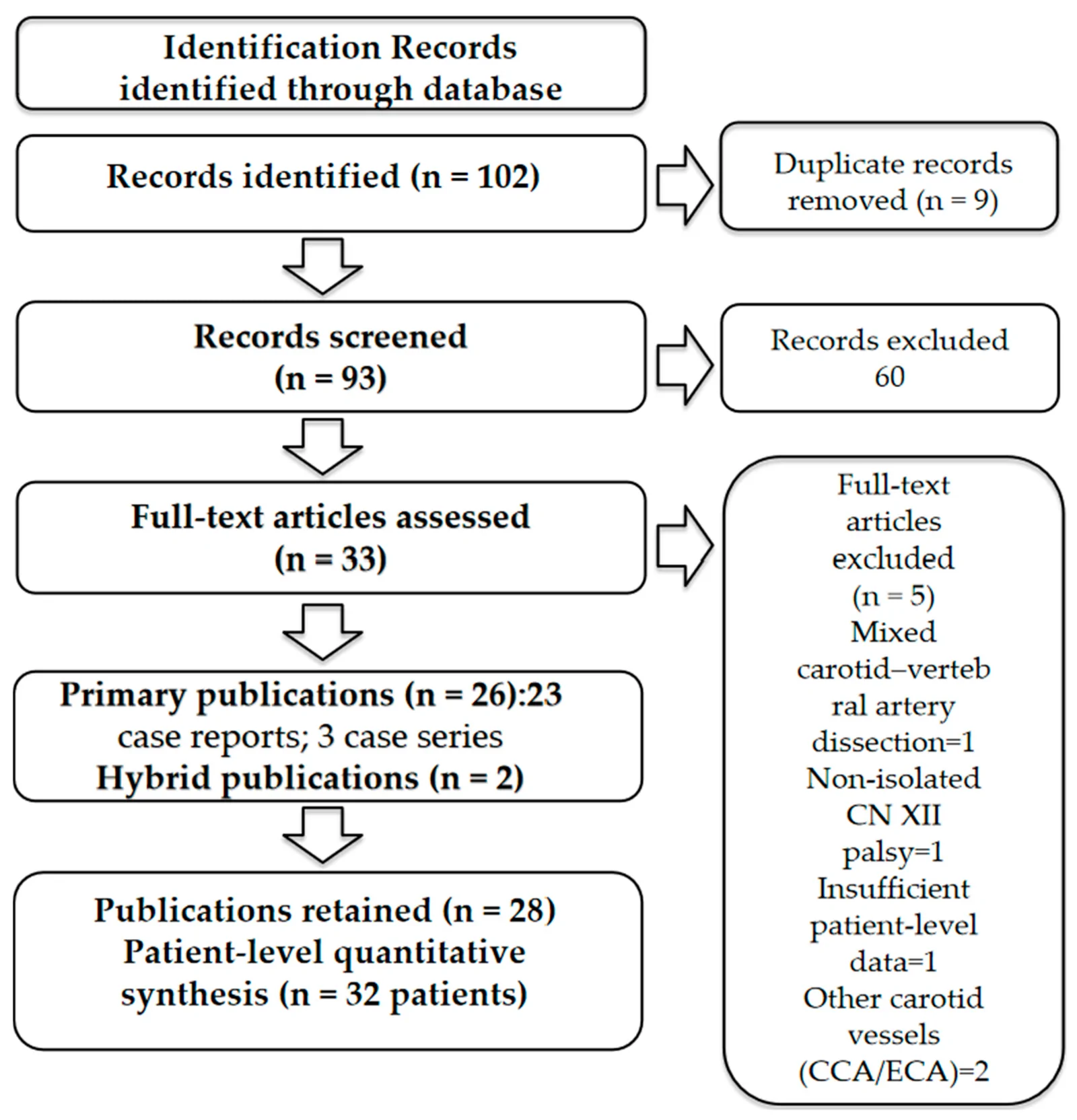
PRISMA 2020 flow diagram of study selection. Overall, 28 publications were retained: 26 standalone primary publications, comprising 23 case reports and 3 case series, and 2 hybrid publications combining original patient reporting with a narrative literature review. Each hybrid publication contributed one eligible patient, resulting in a patient-level quantitative synthesis of 32 patients. The narrative review components were used solely for citation tracking and contextual comparison.

A total of 102 records were identified through database searching. After duplicate removal, 93 records underwent title and abstract screening, resulting in the exclusion of 60 records. The full texts of 33 articles were assessed for eligibility, and 5 were excluded according to the predefined criteria. Overall, 28 publications were retained: 26 standalone primary publications, comprising 23 case reports and 3 case series, and 2 hybrid publications combining original patient reporting with a narrative literature review. Each hybrid publication contributed one eligible patient to the patient-level quantitative synthesis. Mes et al. contained an original case-report component. Sturzenegger and Huber reported two original cases; therefore, their publication was considered to contain a case-series component. Overall, the patient-level quantitative synthesis comprised 32 eligible patients. The narrative review components were used solely for reference-list screening, citation tracking, and contextual comparison and did not contribute secondary patient data to the quantitative synthesis (Figure 1).

Because not all studies reported every variable, percentages were calculated using the number of evaluable patients for each specific variable rather than the total number of included patients. Continuous variables describing follow-up duration were summarized descriptively using the median, mean, and range.

The methodological quality of the original patient-reporting components was assessed using the Joanna Briggs Institute (JBI) critical appraisal tools according to study design. The 23 standalone case reports and the original case-report component of Mes et al. were evaluated using the JBI Critical Appraisal Checklist for Case Reports. The 3 standalone case series and the original case-series component of Sturzenegger and Huber were evaluated using the JBI Critical Appraisal Checklist for Case Series. The narrative review components of the two hybrid publications were separately evaluated using the JBI Critical Appraisal Checklist for Textual Evidence: Narrative. Each checklist item was rated as “Yes”, “No”, “Unclear”, or “Not Applicable”, according to JBI guidance. For descriptive scoring, one point was assigned to each “Yes” response, whereas “No”, “Unclear”, and “Not Applicable” received no points. The maximum possible score was 8 for case reports, 10 for case series, and 6 for narrative evidence. The results of the methodological appraisal are presented in Supplementary Table S3A–C.

Because of the marked heterogeneity in study design, clinical presentation, imaging findings, treatment strategies, and outcome measures, quantitative meta-analysis was not feasible. Therefore, the findings were synthesized descriptively.

### Artificial Intelligence Statement

During the preparation and revision of this manuscript, SciSpace (continuously updated online version; Typeset, Inc., San Francisco, CA, USA) was employed as an AI-assisted tool for language and editorial support, including improvement of textual clarity and organization. The English-language editing process was facilitated by the utilization of DeepL Translator for Windows (version 26.7.2; DeepL SE, Cologne, Germany), with the objective of enhancing grammar, readability, and linguistic quality. Python (version 3.12.13), with Matplotlib (version 3.10.0) and NumPy (version 2.0.2), was executed in Google Colaboratory (Google LLC, Mountain View, CA, USA; cloud-based platform; accessed on 5 August 2026) solely to prepare the schematic figure. It is important to note that no AI-assisted tool was used autonomously for study selection, data extraction, statistical analysis, interpretation of results, or scientific decision-making. Furthermore, no scientific data, references, results, or conclusions were generated by these tools. The source articles, extracted data, numerical values, references, methodological decisions, interpretations, and final wording were independently verified and approved by the authors, who accept full responsibility for the accuracy, integrity, and final content of the manuscript.

## 3. Results

### 3.1. Systematic Review

The literature search identified 102 records. After duplicate removal, 93 records underwent title and abstract screening. Following full-text assessment, 28 publications were retained: 26 standalone primary publications, comprising 23 case reports and 3 case series, and 2 hybrid publications combining original patient reporting with a narrative literature review. Each hybrid publication contributed one eligible patient, resulting in a patient-level quantitative synthesis of 32 patients. The original component of Mes et al. was appraised using the JBI Critical Appraisal Checklist for Case Reports. Because Sturzenegger and Huber reported two original patients, its original component was appraised using the JBI Critical Appraisal Checklist for Case Series, although only one patient fulfilled the eligibility criteria. The narrative review components were separately appraised using the JBI Critical Appraisal Checklist for Textual Evidence: Narrative and were used solely for citation tracking and contextual comparison. The study-selection process is summarized in the PRISMA 2020 flow diagram (Figure 1). The original patient-reporting components fulfilled most of the applicable JBI critical appraisal items, with incomplete follow-up reporting and limited information on predisposing factors representing the most common methodological limitations. Both narrative review components achieved a score of 6/6 and were considered appropriate for contextual comparison (Supplementary Table S3A–C).

### 3.2. Clinical Presentation and Diagnostic Assessment

Of the 32 patients, 28 (87.5%) were male, and 4 (12.5%) were female, with a median age of 47 years. Hypertension was the most frequently reported vascular risk factor, occurring in 9 of 20 evaluable patients (45.0%). Smoking status was available for only five patients, three of whom were smokers. Antecedent events preceding symptom onset included minor cervical mechanical triggers (7/26, 26.9%), trauma (3/26, 11.5%), dental procedures (3/11, 27.3%), recent infection (3/6, 50.0%), vigorous cough or Valsalva maneuver (1/8, 12.5%), chiropractic manipulation (1/13, 7.7%), connective tissue disorder or genetic disease (1/4, 25.0%), and other vascular risk factors (3/13, 23.1%). No identifiable triggering event was reported in 17 of 25 evaluable patients (68.0%). The demographic characteristics and predisposing factors of the included patients are summarized in Table 1.

Tongue deviation was reported in all evaluable patients (28/28, 100%), while tongue weakness was documented in all evaluable patients (30/30, 100%). Dysarthria occurred in all evaluable patients (26/26, 100%), followed by dysphagia in 18 of 22 evaluable patients (81.8%). Headache or retro-orbital pain was reported in 12 of 18 evaluable patients (66.7%), neck pain in 8 of 10 (80.0%), mandibular pain in 5 of 7 (71.4%), and facial pain in 4 of 5 (80.0%). Tongue atrophy was described in all evaluable patients (10/10, 100%), whereas MRI evidence of tongue denervation was documented in 8 of 9 evaluable patients (88.9%). Tongue swelling or pseudohypertrophy was reported in 9 of 9 evaluable patients (100%), and tongue fasciculations in 2 of 2 evaluable patients (100%). Isolated hypoglossal nerve palsy was present in all 32 patients (100%). Delayed glossopharyngeal nerve (CN IX) involvement occurred in one patient (3.1%), whereas no patient presented with concomitant vagal or accessory nerve palsy, Horner syndrome, Collet–Sicard syndrome, or Villaret syndrome.

MRI was the most frequently reported imaging modality, being performed in 29 of 32 patients (90.6%), followed by CT (19/24 evaluable patients, 79.2%), MRA (17/24, 70.8%), CTA (13/18, 72.2%), carotid ultrasound (16/24, 66.7%), and digital subtraction angiography (DSA) (7/19, 36.8%) (Table 2). Left-sided ICAD was more common than right-sided disease (24/32 [75.0%] vs. 5/32 [15.6%]), whereas bilateral involvement was observed in 3 of 32 patients (9.4%).

Among evaluable patients, intramural hematoma was identified in 25 of 26 cases (96.2%), luminal stenosis in 23 of 26 (88.5%), and external arterial enlargement in 22 of 24 (91.7%). Mass effect on the hypoglossal nerve was reported in 19 of 22 evaluable patients (86.4%), whereas direct radiological evidence of CN XII compression was documented in 3 of 32 patients (9.4%). Pseudoaneurysm or dissecting aneurysm was described in 8 of 21 evaluable patients (38.1%), internal carotid artery occlusion in 5 of 28 (17.9%), and direct evidence of a double lumen was described in only four patients (4/4 evaluable cases). A classic string sign was documented in two patients, whereas one additional case described abrupt tapering without explicitly using the term “string sign”. Neuroimaging findings are summarized in Table 2.

Conservative medical management was the predominant therapeutic approach, reported in 31 of the 32 patients (96.9%). Information on antithrombotic therapy was available for 31 patients. Among these, antiplatelet therapy was administered in 19 (61.3%), anticoagulation in 14 (45.2%), and dual antiplatelet therapy in 5 (16.1%). Endovascular treatment was performed in 2 of the 32 patients (6.3%), whereas no patient underwent surgical treatment.

Clinical outcome data were available for 30 of the 32 patients. Complete recovery was observed in 17 patients (56.7%), marked clinical improvement in 4 (13.3%), partial recovery or persistent neurological deficit in 7 (23.3%), and stable neurological status without deterioration in 2 (6.7%). Clinical outcomes were not reported for the remaining two patients. Overall, clinical improvement of any degree was reported in 25 of the 30 evaluable patients (83.3%). Imaging follow-up was performed in 18 of the 32 patients (56.3%). Among these, radiological outcome data were available for 16 patients. Complete radiological resolution was reported in 7 of 16 patients (43.8%), partial radiological improvement in 6 (37.5%), and persistent vascular abnormalities in 10 (62.5%). These categories were not mutually exclusive, as radiological improvement could occur despite residual vascular abnormalities. No recurrence of internal carotid artery dissection was reported among the seven patients for whom recurrence data were available. The median clinical follow-up was approximately 3 months, with a mean follow-up of approximately 5.3 months (range, 0.5–36 months). Treatment outcomes are summarized in Table 3.

### 3.3. Review Articles

Two hybrid publications combined original patient reporting with a narrative review of the literature. Mes et al. reported one original case, which fulfilled the eligibility criteria and was included in the patient-level quantitative synthesis. Its original component was appraised using the JBI Critical Appraisal Checklist for Case Reports. Sturzenegger and Huber reported two original cases and were therefore considered to contain a case-series component; however, only one of these patients fulfilled the eligibility criteria and contributed to the patient-level quantitative synthesis. Its original component was appraised using the JBI Critical Appraisal Checklist for Case Series. The narrative review components of both publications were excluded from the quantitative synthesis, separately appraised using the JBI Critical Appraisal Checklist for Textual Evidence: Narrative, and used solely for citation tracking and contextual comparison with previous literature.

Sturzenegger and Huber (1993) [14] reviewed 36 previously published cases and presented two new cases, bringing the total to 38. Of these, 27 presented with hypoglossal nerve involvement, including ten with isolated hypoglossal nerve palsy. Of these, one of the two original cases fulfilled the eligibility criteria of the present systematic review and was therefore included in the quantitative synthesis. Of the 36 cases summarized in the literature by the authors, only four met the predefined eligibility criteria of the present review (isolated hypoglossal nerve palsy associated with ICAD without Horner syndrome). These four cases were analyzed descriptively, as the review provided insufficient patient-level information for quantitative extraction, and potential duplication with the original reports could not be excluded.

The four eligible cases (three men and one woman; mean age 46 years) presented with left-sided ICAD, isolated hypoglossal nerve palsy, tongue deviation and mild dysarthria or tongue dysfunction. None of the patients presented with Horner syndrome, additional cranial nerve involvement or cerebral ischemia. Diagnosis was established by digital subtraction angiography, complemented by CT and MRI in the more recent cases. MRI scans revealed intramural haematoma, luminal stenosis, pseudoaneurysm and arterial wall enlargement. All patients were managed conservatively with anticoagulation, and clinical recovery was reported in every case. When available, follow-up imaging showed progressive or complete resolution of the intramural haematoma, alongside recovery of hypoglossal nerve function.

Mes et al. (2018) [15] published the first review specifically dedicated to isolated hypoglossal nerve palsy associated with ICAD. They identified 22 previously published cases and reported one additional original case, which is also included in the present systematic review. Table 4 compares the methodological characteristics and principal clinicoradiological findings reported by Mes et al. with those of the present systematic review.

In comparison with the preceding review by Mes et al., the present review offers an updated synthesis of the extant evidence up to April 2026 and incorporates a more substantial pooled cohort of eligible patients (32 versus 22). Mes et al. [15] identified 18 primary publications; however, three reports (Mateen et al. [13], Bezerra et al. [27], and Hennings et al. [28]) were not eligible according to the predefined inclusion criteria of the present review, which required isolated hypoglossal nerve palsy secondary to isolated internal carotid artery dissection. Consequently, a total of 15 standalone primary publications were shared between the two reviews, while the updated systematic search identified 11 additional eligible standalone primary publications, resulting in a total of 26 standalone primary publications. Among these, Jurkiewicz et al. [26] contributed four eligible patients and Olzowy et al. contributed two. Torbus-Paluszczak et al. [29] reported on two patients, but only one of these met the inclusion criteria and was included in the quantitative synthesis. Overall, the 26 standalone primary publications contributed 30 eligible patients. As a result of the analysis of two hybrid publications by. and Sturzenegger and Huber, it was possible to extract one additional eligible original patient from each of the publications, thus bringing the total pooled cohort to 32 patients. The re-evaluation of all eligible original patient data was conducted using predefined eligibility criteria, with the data then extracted in a standardized manner. This standardization was applied to clinical presentation, neuroradiological findings, treatment, outcomes, and follow-up. This approach enabled a more consistent descriptive synthesis across reports. The narrative review components of Mes et al. and Sturzenegger and Huber were used separately for citation tracking and contextual comparison, and did not contribute secondary patient data to the quantitative synthesis.

## 4. Discussion

Isolated hypoglossal nerve palsy is an uncommon clinical presentation with a broad differential diagnosis, in which neoplastic disorders remain the most frequent cause. However, this review confirms that internal carotid artery dissection (ICAD) should also be considered, particularly in patients presenting with acute tongue deviation without evidence of cerebral ischemia, Horner syndrome, or other lower cranial nerve deficits [30,31,32,33]. In the present cohort, tongue deviation was documented in all 28 evaluable patients in whom this finding was reported, representing the defining clinical manifestation of ICAD-related hypoglossal neuropathy. Likewise, tongue weakness and dysarthria were consistently reported whenever these variables were described, whereas dysphagia was observed in 18 of the 22 evaluable patients. Headache or retro-orbital pain, neck pain, and mandibular pain were also frequently reported among patients for whom these symptoms were documented. However, these variables were inconsistently reported across the included studies and should therefore be interpreted with caution. Collectively, these findings indicate that lower cranial nerve dysfunction may dominate the initial clinical presentation, potentially delaying recognition of the underlying vascular disorder. Unlike previous reviews addressing the broader spectrum of cranial nerve involvement in ICAD, the present study focused exclusively on isolated hypoglossal nerve palsy. Consequently, patients presenting with Horner syndrome, Collet–Sicard syndrome, Villaret syndrome, or multiple cranial nerve palsies were excluded according to the predefined eligibility criteria. Therefore, the absence of these manifestations in our cohort reflects the study design rather than their true frequency in ICAD [34,35,36,37].

Previous reviews, particularly that by Mes et al., established the association between isolated hypoglossal nerve palsy and internal carotid artery dissection by summarizing the available case reports. Building upon this evidence, the present review expands the available cohort from 22 to 32 patients and provides a comprehensive quantitative characterization of the recurrent clinicoradiological features of this uncommon presentation (Table 4). Specifically, our findings identify a consistent clinical phenotype, a predominant neuroradiological pattern supporting a mainly subadventitial compressive mechanism, and a frequent dissociation between neurological recovery and complete arterial remodelling. The 11 additional primary studies substantially expanded the available neuroradiological evidence, enabling quantitative characterization of recurrent imaging findings—including intramural hematoma, external arterial enlargement, mass effect, pseudoaneurysm formation, and direct hypoglossal nerve compression—which were previously described mainly in isolated case reports. These findings provide a more robust clinicoradiological framework for recognizing this uncommon presentation and are discussed in detail in the following sections.

Although the reviewed patients showed a relatively homogeneous clinical presentation, this profile largely reflects the typical characteristics of spontaneous ICAD, rather than a clinical pattern unique to hypoglossal nerve involvement. The most distinctive feature of this cohort was the frequent absence of cerebral ischemia and other neurological deficits, despite the presence of carotid artery dissection. Clinically, this finding has important implications because patients with isolated tongue dysfunction may initially be referred to otolaryngology, oral medicine, maxillofacial surgery, or general neurology, rather than to stroke services.

An additional diagnostic consideration concerns the interpretation of acute tongue deviation. In supranuclear stroke, tongue deviation typically occurs contralateral to the cerebral lesion, whereas ICAD-related hypoglossal neuropathy produces an ipsilateral lower motor neuron deficit. Recognizing this distinction, together with awareness that intramural hematoma may occasionally be identifiable retrospectively on initial non-contrast CT examinations, may facilitate earlier consideration of vascular imaging and reduce diagnostic delay.

Two principal mechanisms have been proposed to explain hypoglossal nerve dysfunction in ICAD: direct mechanical compression caused by outward expansion of the dissected arterial wall and ischaemic injury involving the vasa nervorum. Based on the available case-level evidence, both mechanisms remain biologically plausible, although the published neuroradiological findings appear more frequently consistent with a compressive process [38,39,40].

A useful conceptual distinction can be made between subintimal and subadventitial dissections. In subintimal dissections, mural hematoma expands towards the vascular lumen, producing stenosis or occlusion and increasing the likelihood of cerebral ischemia. In contrast, subadventitial dissections are characterized by outward expansion of the arterial wall, often with limited luminal compromise, resulting in close anatomical proximity to adjacent lower cranial nerves.

Across the reviewed cases, several neuroradiological observations were compatible with this mechanism. These included mural hematoma extending into the parapharyngeal or retrostyloid space, enlargement of the internal arterial diameter, and close anatomical contact between the dissected internal carotid artery and the hypoglossal nerve. In a minority of reports, the nerve itself was displaced or no longer identifiable because of mass effect from the expanded arterial wall. Collectively, these observations are consistent with, but do not definitively establish, direct mechanical compression as a contributor to hypoglossal nerve dysfunction.

Intramural hematoma was the most consistently reported imaging finding, whereas luminal stenosis and external arterial enlargement were also frequently observed. Pseudoaneurysm formation, the double-lumen sign, and the string sign were reported less commonly but, when present, provided additional evidence supporting the diagnosis of ICAD. The variability in the frequency of these findings likely reflects differences in lesion location, imaging protocols, and timing of image acquisition, rather than true biological variability.

Overall, the available evidence suggests that hypoglossal nerve palsy associated with ICAD is compatible with a predominantly subadventitial pattern of dissection in many reported cases. Nevertheless, because direct radiological evidence of nerve compression was documented only in a subset of patients and imaging protocols were heterogeneous, an ischaemic contribution through involvement of the vasa nervorum cannot be excluded. Further studies using dedicated, high-resolution vessel-wall imaging are required to clarify the relative contribution of these mechanisms.

Neuroradiological assessment represents a central component of the diagnostic evaluation of ICAD-associated isolated hypoglossal nerve palsy. MRI was the most frequently reported imaging modality, being performed in 29 of the 32 patients (90.6%). MRA and CTA were also commonly used when reported, although their apparent frequencies reflect incomplete reporting rather than true utilization rates. This distribution likely reflects both the historical evolution of imaging techniques and the ability of MRI to directly demonstrate vessel-wall abnormalities, including intramural hematoma and external arterial enlargement.

Across the reviewed literature, MRI—particularly fat-suppressed T1-weighted sequences—was the modality most commonly used to identify intramural hematoma, arterial wall enlargement, and the anatomical relationship between the dissected internal carotid artery and the hypoglossal nerve. T2-weighted sequences additionally demonstrated the longitudinal extension of mural haematoma and, in selected cases, secondary denervation changes within the tongue. More recently, vessel-wall MRI and balanced steady-state free precession (TrueFISP) sequences have been reported as useful adjuncts for evaluating the vessel wall and its relationship to adjacent cranial nerves.

CTA primarily provided information regarding luminal abnormalities, including stenosis, occlusion, intimal flap, double-lumen appearance, and pseudoaneurysm formation. Because it is widely available in the emergency setting and allows rapid assessment of both the carotid and vertebral arteries, CTA remains an important imaging modality when a vascular cause is suspected.

Rather than representing competing techniques, MRI/MRA and CTA provide complementary diagnostic information. MRI/MRA is particularly suited for assessing vessel-wall pathology, intramural hematoma, and the anatomical relationship between the dissected artery and the hypoglossal nerve, whereas CTA rapidly characterizes luminal abnormalities and associated vascular complications. Accordingly, the choice and sequence of imaging should be guided by the clinical presentation, diagnostic urgency, local availability, contraindications, and institutional expertise rather than by a fixed diagnostic strategy.

Digital subtraction angiography was reported only in selected patients, mainly when diagnostic uncertainty persisted, or endovascular treatment was considered. Although it remains the reference standard for assessment of the arterial lumen, it provides limited information regarding mural hematoma and perivascular soft tissues. Similarly, carotid Doppler ultrasound demonstrated lower diagnostic sensitivity, particularly for high cervical or skull-base dissections, and several reports described normal or inconclusive ultrasound examinations, subsequently followed by diagnostic MRI or CTA.

Electrophysiological assessment was infrequently reported. When performed, electromyography confirmed lower motor neuron hypoglossal neuropathy by demonstrating spontaneous denervation activity and reduced motor unit recruitment. Although these findings support the diagnosis of hypoglossal nerve dysfunction, they do not identify its vascular cause, and should therefore be considered complementary to, rather than a substitute for, neurovascular imaging.

MRI may also demonstrate indirect evidence of chronic hypoglossal denervation. Acute denervation may manifest as T2 hyperintensity due to muscular edema, whereas chronic denervation is characterized by progressive fatty replacement and hemiatrophy, with increased T1 signal intensity. In patients presenting several weeks or months after symptom onset, these secondary muscular changes may represent the most conspicuous imaging abnormality and should prompt careful evaluation of the cervical internal carotid artery for evidence of previous dissection.

Overall, the available literature indicates that no single imaging modality is sufficient to characterize all aspects of ICAD-associated hypoglossal nerve palsy. Instead, CTA and MRI/MRA should be regarded as complementary investigations, each providing distinct but clinically relevant information that contributes to the diagnosis according to the individual clinical context.

Conservative medical management was the predominant therapeutic approach and was reported in 31 of the 32 patients (96.9%). Among the 31 patients for whom antithrombotic treatment was described, antiplatelet therapy was administered in 19 (61.3%), anticoagulation in 14 (45.2%), and dual antiplatelet therapy in 5 (16.1%). Endovascular intervention was reserved for selected cases, including enlarging pseudoaneurysms or persistent symptoms despite medical treatment.

The available literature does not demonstrate the superiority of either antiplatelet therapy or anticoagulation for patients presenting with isolated hypoglossal nerve palsy secondary to ICAD. Consequently, treatment decisions should follow current recommendations for cervical artery dissection and be individualized according to the overall clinical and neuroradiological presentation rather than the presence of hypoglossal nerve palsy alone. The findings of the CADISS trial support the use of either therapeutic strategy in appropriately selected patients [41,42,43,44].

Clinical outcome data were available for 30 of the 32 patients. Complete recovery was reported in 17 patients, while a further four experienced marked clinical improvement, corresponding to a favourable clinical outcome in 21 of the 30 evaluable patients (70.0%). Overall, improvement of any degree, including partial recovery, was documented in 25 of the 30 evaluable patients (83.3%). Nevertheless, these findings should be interpreted descriptively rather than as reliable estimates of prognosis. The available evidence is derived almost exclusively from isolated case reports and small case series and is therefore susceptible to publication bias, selective reporting, heterogeneous and generally short follow-up, and non-standardized outcome assessment. The median clinical follow-up was approximately 3 months, although follow-up duration varied considerably across reports (range, 0.5–36 months), potentially leading to underestimation of delayed neurological recovery or late vascular remodelling. Patients with favourable outcomes are also more likely to be published than those with persistent neurological deficits.

Despite these limitations, most published reports described clinical improvement following conservative management, suggesting that neurological recovery is possible in many patients when ICAD is recognized and appropriately treated. Nevertheless, the available evidence remains insufficient to define prognostic factors or to compare the effectiveness of different therapeutic strategies, highlighting the need for prospective, multicentre studies with standardized clinical and radiological follow-up.

One of the principal findings of the present review is the apparent dissociation between clinical and radiological recovery. While a favourable neurological outcome was observed in 21 of the 30 evaluable patients (70.0%), persistent vascular abnormalities were still present in 10 of the 16 patients (62.5%) who underwent follow-up imaging. These findings suggest that neurological recovery may occur despite incomplete arterial healing. A possible explanation is that reduction in the intramural hematoma and the associated mass effect is sufficient to relieve hypoglossal nerve compression, allowing functional recovery even in the presence of residual arterial abnormalities such as stenosis or pseudoaneurysm. Consequently, persistent vascular abnormalities on follow-up imaging should not necessarily be interpreted as indicators of poor neurological prognosis. Nevertheless, this interpretation should be considered hypothesis-generating, as radiological follow-up was available in only 16 patients and was heterogeneous in terms of timing and imaging protocols.

## 5. Conclusions

This systematic review provides the first standardized synthesis of the available case-level evidence on isolated hypoglossal nerve palsy secondary to internal carotid artery dissection. Despite the very low level of evidence, it identifies a consistent clinicoradiological profile that may facilitate earlier recognition of this uncommon presentation, particularly in emergency settings where isolated hypoglossal nerve palsy may not immediately raise suspicion for carotid artery dissection. The findings support the complementary role of MRI/MRA and CTA in the diagnostic work-up and suggest that prompt vascular imaging may enable timely initiation of appropriate medical management. They also indicate that neurological recovery may occur despite persistent vascular abnormalities, suggesting that clinical recovery and vascular remodelling do not necessarily occur in parallel. Although definitive diagnostic or therapeutic recommendations cannot be drawn, this review provides a practical framework for early diagnosis and highlights the need for prospective multicentre studies using standardized imaging protocols, validated outcome measures, and longer-term clinical and radiological follow-up.

## Figures and Tables

**Table 1:** Demographic Characteristics and Predisposing Factors of Included Patients (*n* = 32).

**Variable**	**n/N (%)**
Male sex	28/32 (87.5%)
Female sex	4/32 (12.5%)
Median age	47 years
Medical history/vascular risk factors	n/N (%)
Hypertension	9/20 (45.0%)
Smoking	3/5 (60.0%)
**Antecedent Events Before Symptom Onset**	**n/N (%)**
Minor cervical mechanical events *	7/26 (26.9%)
Vigorous cough/Valsalva	1/8 (12.5%)
Trauma	3/26 (11.5%)
Recent infection/pharyngitis	3/6 (50.0%)
Dental procedure	3/11 (27.3%)
Chiropractic manipulation	1/13 (7.7%)
Genetic predisposition/(PKD1 mutation)	1/4 (25.0%)
Other vascular risk factors	3/13 (23.1%)
No identifiable trigger	17/25 (68.0%)

* Minor cervical mechanical triggers included activities such as sports, sudden neck movement, cervical manipulation (excluding chiropractic manipulation), heavy lifting, coughing, or other minor mechanical events preceding symptom onset.

**Table 2:** Neuroimaging Characteristics of Included Patients (*n* = 32).

**Imaging Modalities**	**n/N (%)**
Magnetic resonance imaging (MRI)	29/32 (90.6%)
Magnetic resonance angiography (MRA)	17/24 (70.8%)
Computed tomography angiography (CTA)	13/18 (72.2%)
Computed tomography (CT)	19/24 (79.2%)
Carotid ultrasound	16/24 (66.7%)
Digital subtraction angiography (DSA)	7/19 (36.8%)
**Radiological Findings**	**n/N (%)**
Left ICA	24/32 (75.0%)
Right ICA	5/32 (15.6%)
Bilateral ICA	3/32 (9.4%)
Intramural hematoma	25/26 (96.2%)
Luminal stenosis	23/26 (88.5%)
External arterial enlargement	22/24 (91.7%)
Mass effect on the HN-p	19/22 (86.4%)
CN XII compression	3/32 (9.4%)
Pseudoaneurysm/dissecting aneurysm	8/21 (38.1%)
ICA occlusion	5/28 (17.9%)
String sign or string-like tapering	3/29 (10.3%) †

Note: Radiological findings were not mutually exclusive, and individual patients could present with multiple imaging features of internal carotid artery dissection. † The string sign was explicitly reported in only two cases; one additional case described abrupt tapering without explicitly using the term “string sign”.

**Table 3:** Treatment and Clinical Outcomes of Included Patients (*n* = 32).

**Treatment**	**n/N (%)**
Conservative treatment	31/32 (96.9%)
Antiplatelet therapy	19/31 (61.3%)
Dual antiplatelet therapy	5/31 (16.1%)
Anticoagulation	14/31 (45.2%)
Endovascular treatment	2/32 (6.3%)
Surgical treatment	0/32 (0%)
**Outcome**	**n/N (%)**
Complete recovery	17/30 (56.7%)
Marked improvement	4/30 (13.3%)
Incomplete recovery	7/30 (23.3%)
Stable/no deterioration	2/30 (6.7%
Outcome not reported	2/32 (6.3%)

Note: Treatment categories were not mutually exclusive, as some patients received sequential antithrombotic therapy (e.g., anticoagulation followed by antiplatelet therapy) during the course of management.

**Table 4:** Methodological and clinicoradiological comparison between the review by Mes et al. (2018) [15] and the present systematic review.

Feature	Mes et al.	Systematic Review
Methodology	Narrative review	PRISMA 2020/JBI critical
Publications	18 (15 eligible)	26 (+11; +73%) [16,17,18,19,20,21,22,23,24,25,26]
Patients included	22	32 (+10; +45.5%)
Neuroradiological findings	Descriptive	Recurrent clinicoradiological profile (TW/TD + IH, EAE, ME, PA)
Mechanism of CN XII palsy	Mainly discussed	Imaging-supported compressive mechanism
Clinical–radiological evolution	Not specifically	Discordance between clinical recovery and radiological resolution highlighted
Outcome analysis	Descriptive	Clinical recovery and PVA systematically assessed

Note: IH, intramural hematoma; EAE, external arterial enlargement; ME, mass effect; PA, pseudoaneurysm; PVA, persistent vascular abnormalities. TW, Tongue Weakness; TD, Tongue Deviation.

## Data Availability

All data analyzed during this study are included in the published article. No new datasets were generated.

## Supplementary Materials

The following supporting information can be downloaded at: https://www.mdpi.com/article/10.3390/neurolint18080150/s1. Supplementary File S1: PRISMA 2020 Checklist. Supplementary Table S1: Detailed search strategy for PubMed, Scopus, Web of Science, and Cochrane Library databases. Supplementary Table S2: Individual patient characteristics, imaging findings, treatment, and outcomes of the included studies. Supplementary Table S3A: JBI critical appraisal of the included case reports; Supplementary Table S3B: JBI critical appraisal of the included case series; Supplementary Table S3C: JBI critical appraisal of the narrative review components.

## Abbreviations

The following abbreviations are used in this manuscript:

ICAD	Internal Carotid Artery Dissection
HN p	Hypoglossal Nerve palsy
CT	Computed Tomography
CTA	Computed Tomography Angiography
CTP	Computed Tomography Perfusion
MRI	Magnetic Resonance Imaging
DSA	Digital Subtraction Angiography
CN XII	Twelfth Cranial Nerve (Hypoglossal Nerve)
MRA	Magnetic Resonance Angiography
JBI	Joanna Briggs Institute
PRISMA	Preferred Reporting Items for Systematic Reviews and Meta-Analyses
PROSPERO	International Prospective Register of Systematic Reviews
TOF-MRA	Time-of-Flight Magnetic Resonance Angiography
TrueFISP	True Fast Imaging with Steady-State Precession
HRMRI	High-Resolution Magnetic Resonance Imaging
PKD1	Polycystic Kidney Disease
IH	Intramural hematoma
EAE	External arterial enlargement
ME	Mass effect
PA	Pseudoaneurysm
PVA	Persistent vascular abnormalities
TW	Tongue Weakness
TD	Tongue Deviation
