# Supplementary material for: Isolated Hypoglossal Nerve Palsy Associated with Internal Carotid Artery Dissection: A Systematic Review
Source: Neurol Int. 2026 Aug 14;18(8):150. doi: 10.3390/neurolint18080150 (PMC13517042; doi:10.3390/neurolint18080150)
Supplement: Supplementary file 1 [file neurolint-18-00150-s001.zip › Supplementary Table S1.pdf]

Supplementary Material

Supplementary Table S1. Detailed search strategy for PubMed, Scopus, Web of Science, and Cochrane Library databases

Database	Search string
PubMed	("internal carotid artery dissection" OR "carotid artery dissection") AND ("hypoglossal nerve palsy" OR "hypoglossal nerve")
Scopus	TITLE-ABS-KEY ("internal carotid artery dissection" OR "carotid artery dissection") AND TITLE-ABS-KEY ("hypoglossal nerve palsy" OR "hypoglossal nerve")
Cochrane Library	("internal carotid artery dissection" OR "carotid artery dissection"):ti,ab,kw AND ("hypoglossal nerve palsy" OR "hypoglossal nerve"):ti,ab,kw
Web of Science	TS=("internal carotid artery dissection" OR "carotid artery dissection") AND TS=("hypoglossal nerve palsy" OR "hypoglossal nerve")
