# Supplementary material for: Isolated Hypoglossal Nerve Palsy Associated with Internal Carotid Artery Dissection: A Systematic Review
Source: Neurol Int. 2026 Aug 14;18(8):150. doi: 10.3390/neurolint18080150 (PMC13517042; doi:10.3390/neurolint18080150)
Supplement: Supplementary file 1 [file neurolint-18-00150-s001.zip › Supplementary Table S2.pdf]

Table S2. Individual patient characteristics, imaging findings, treatment, and outcomes of the included studies

Freilinger et al., 2010

Title: *Hypoglossal nerve palsy due to internal carotid artery dissection*

Authors: Tobias Freilinger, Andreas Heuck, Michael Strupp, Rainer Jund

Journal: *Vascular Medicine*

Year: 2010

Study design: Case report

Table 4. Demographic and Etiopathogenetic Characteristics

Variable	Finding
Age / Sex	**35 years / Male**
Smoking	NR
Hypertension	NR
Connective tissue disorder / genetic disease	NR
Other vascular risk factors	None reported
Trauma	**Minor trauma (judo training)**
Minor cervical mechanical trigger	**Yes (judo training)**
Recent infection	No
Dental procedure	No
Chiropractic manipulation	No
Vigorous cough / Valsalva	No
Other predisposing factor	None reported

Table 5. Clinical and Imaging Characteristics

Variable	Finding
Internal carotid artery dissection	**Yes (Right ICA)**
Time from symptom onset to diagnosis	NR
Headache	No
Neck pain	**Yes (right neck pain)**
Mandibular pain	No
Facial pain	No

Variable	Finding
Dysarthria	**Yes (articulation problems)**
Dysphagia	No
Tongue deviation	**Yes (right)**
Tongue weakness	**Yes**
Tongue atrophy	No
Tongue swelling / pseudohypertrophy	No
Fasciculations	NR
Isolated CN XII palsy	**Yes**
Other cranial nerve involvement	No
Collet–Sicard syndrome	No
Horner syndrome	No
Stroke / cerebral ischemia	No (brain MRI negative for infarction)
Initial misdiagnosis	No
First specialty consulted	**ENT specialist**
MRI	**Yes**
MRA	No
CTA	No
CT	No
Doppler ultrasound	No
Duplex ultrasound	**Yes**
DSA	No
Intramural hematoma	**Yes**
Double lumen	No
Intimal tear	NR
Luminal stenosis	**Yes** (without hemodynamically relevant stenosis on duplex)
ICA occlusion	No
String sign	No
External arterial enlargement	**Yes (subadventitial mural expansion)**
Pseudoaneurysm / dissecting aneurysm	No
Direct CN XII compression demonstrated	**Yes**

Variable	Finding
Mass effect on CN XII	**Yes**

Table 6. Treatment and Outcome Characteristics

Variable	Finding
Conservative treatment	**Yes**
Antiplatelet therapy	**Yes (aspirin after anticoagulation)**
Anticoagulation	**Yes (oral anticoagulation for 6 months)**
Dual antiplatelet therapy	No
Endovascular treatment	No
Surgical treatment	No
Treatment duration	**Oral anticoagulation for 6 months, then aspirin**
Clinical outcome	**Complete clinical recovery**
Follow-up duration	A few weeks (clinical recovery)
Imaging follow-up	NR
Complete radiological resolution	NR
Partial radiological improvement	NR
Persistent vascular abnormality	NR
Recurrence	No reported

Table 1

- Uomo
- 35 anni
- Trigger meccanico cervicale (judo)
- Nessuna ipertensione riportata
- Nessun fumo riportato
- Dolore cervicale
- Disartria
- Deviazione della lingua
- Paralisi isolata del XII
- Nessun Horner
- Nessun ictus

Table 2

- MRI

- Duplex
- Ematoma intramurale
- Stenosi del lume
- Espansione subavventiziale
- Compressione diretta del XII dimostrata
- Nessun pseudoaneurisma
- Nessuna ischemia cerebrale

Table 3

- Anticoagulazione
- Aspirina
- Trattamento conservativo
- Recupero completo
- Nessuna recidiva riportat

____-

Allingham et al., 2018

Title: *Hypoglossal Nerve Palsy Due to Internal Carotid Artery Dissection*

Authors: William Allingham, Vinodh Devakumar, Amit Herwadkar, Martin Punter

Journal: *The Neurohospitalist*

Year: 2018

Study design: Case report

Table 4. Demographic and Etiopathogenetic Characteristics

**Variable**	**Finding**
Age / Sex	**40 years / Male**
Smoking	NR
Hypertension	NR
Connective tissue disorder / genetic disease	NR
Other vascular risk factors	None reported
Trauma	No
Minor cervical mechanical trigger	No
Recent infection	No
Dental procedure	No

Variable	Finding
Chiropractic manipulation	No
Vigorous cough / Valsalva	No
Other predisposing factor	None reported

Table 5. Clinical and Imaging Characteristics

Variable	Finding
Internal carotid artery dissection	**Yes (Left ICA)**
Time from symptom onset to diagnosis	**2 weeks (headache), dysarthria developed during the last 5 days**
Headache	**Yes**
Neck pain	No
Mandibular pain	No
Facial pain	No
Dysarthria	**Yes (mild)**
Dysphagia	No
Tongue deviation	**Yes (left)**
Tongue weakness	**Yes**
Tongue atrophy	No
Tongue swelling / pseudohypertrophy	No
Fasciculations	NR
Isolated CN XII palsy	**Yes**
Other cranial nerve involvement	No
Collet–Sicard syndrome	No
Horner syndrome	No
Stroke / cerebral ischemia	No
Initial misdiagnosis	NR
First specialty consulted	NR
MRI	**Yes**
MRA	**Yes (contrast-enhanced MR angiography)**
CTA	No
CT	No

Variable	Finding
Doppler ultrasound	No
Duplex ultrasound	No
DSA	No
Intramural hematoma	**Yes**
Double lumen	NR
Intimal tear	NR
Luminal stenosis	**Yes**
ICA occlusion	No
String sign	No
External arterial enlargement	**Yes (mural expansion)**
Pseudoaneurysm / dissecting aneurysm	**Yes**
Direct CN XII compression demonstrated	**No (hypothesized, not directly demonstrated)**
Mass effect on CN XII	**Suggested**

Table 6. Treatment and Outcome Characteristics

Variable	Finding
Conservative treatment	**Yes**
Antiplatelet therapy	**Aspirin (300 mg for 2 weeks, then 75 mg daily)**
Anticoagulation	No
Dual antiplatelet therapy	No
Endovascular treatment	No
Surgical treatment	No
Treatment duration	**Aspirin 300 mg for 2 weeks, followed by 75 mg daily**
Clinical outcome	**Good clinical recovery with resolution of tongue deviation**
Follow-up duration	**3 months**
Imaging follow-up	**Yes (repeat arterial MR imaging)**
Complete radiological resolution	No
Partial radiological improvement	NR
Persistent vascular abnormality	**Persistent ICA pseudoaneurysm**
Recurrence	No reported

Allingham et al., 2018 scriverei:

Comment: This case is notable for the presence of a persistent internal carotid artery pseudoaneurysm despite complete clinical recovery of the hypoglossal nerve palsy. The authors discuss both compressive and ischemic mechanisms of nerve injury, ultimately favoring ischemia of the *vasa nervorum* because neurological recovery occurred without radiological resolution of the pseudoaneurysm. No ischemic stroke or additional cranial nerve involvement was observed.

>Table 1

- ✓ Male
- ✓ 40 years
- ✓ Headache
- ✓ Dysarthria
- ✓ Isolated CN XII
- ✓ No Horner
- ✓ No stroke

Table 2

- ✓ MRI
- ✓ MRA
- ✓ Intramural hematoma
- ✓ Luminal stenosis
- ✓ Pseudoaneurysm
- ✓ External arterial enlargement
- ✓ **NO** direct compression demonstrated
- ✓ Suggested mass effect
- ✓ No cerebral ischemia

Questo articolo sarà uno dei pochi che contribuirà alla categoria:

Pseudoaneurysm with persistent vascular abnormality

Table 3

- ✓ Aspirin
- ✓ Conservative treatment
- ✓ Complete recovery
- ✓ Imaging follow-up
- ✓ Persistent pseudoaneurysm
- ✓ No recurrence

Jurkiewicz et al., 2019 – Patient 1

Title: *Hypoglossal nerve palsy due to carotid artery dissection: an uncommon presentation of a common problem*

Authors: Michael T. Jurkiewicz, Joel M. Stein, Kim O. Learned, Ilya M. Nasrallah, Laurie A. Loevner

Journal: *The Neuroradiology Journal*

Year: 2019

Study design: Case series (Patient 1 extracted)

Table 4. Demographic and Etiopathogenetic Characteristics

Variable	Finding
Age / Sex	**52 years / Female**
Smoking	NR
Hypertension	NR
Connective tissue disorder / genetic disease	NR
Other vascular risk factors	NR
Trauma	No
Minor cervical mechanical trigger	No
Recent infection	NR
Dental procedure	No
Chiropractic manipulation	No
Vigorous cough / Valsalva	NR
Other predisposing factor	**Spontaneous**

Table 5. Clinical and Imaging Characteristics

Variable	Finding
Internal carotid artery dissection	**Yes (Right ICA)**
Side of ICA dissection	**Right**
Time from symptom onset to diagnosis	**14 days**
Headache	NR
Neck pain	NR
Mandibular pain	NR
Facial pain	NR

**Variable**	**Finding**
Dysarthria	**Yes**
Dysphagia	**Yes**
Tongue deviation	**Yes**
Tongue weakness	**Yes**
Tongue atrophy	**Yes**
Tongue swelling / pseudohypertrophy	NR
Fasciculations	NR
Isolated CN XII palsy	**Yes**
Other cranial nerve involvement	No
Collet–Sicard syndrome	No
Horner syndrome	NR
Stroke / cerebral ischemia	No
Initial misdiagnosis	NR
First specialty consulted	NR
MRI	**Yes**
MRA	**Yes**
CTA	**Yes**
CT	**Yes**
Doppler ultrasound	No
Duplex ultrasound	No
DSA	No
Intramural hematoma	**Yes**
Double lumen	NR
Intimal tear	NR
Luminal stenosis	**>80%**
ICA occlusion	No
String sign	NR
External arterial enlargement	**Yes**
Pseudoaneurysm / dissecting aneurysm	NR
Direct CN XII compression demonstrated	**No**

Variable	Finding
Mass effect on CN XII	**Suggested (adjacent to the hypoglossal canal)**

Table 6. Treatment and Outcome Characteristics

Variable	Finding
Conservative treatment	**Yes**
Antiplatelet therapy	**Yes**
Anticoagulation	NR
Dual antiplatelet therapy	NR
Endovascular treatment	No
Surgical treatment	No
Treatment duration	NR
Clinical outcome	**Improved tongue function and swallowing**
Follow-up duration	**2 weeks**
Imaging follow-up	NR
Complete radiological resolution	NR
Partial radiological improvement	NR
Persistent vascular abnormality	NR
Recurrence	NR

Patient 2

Table 4

Variable	Finding
**Age / Sex**	**46 years / Male**
**Internal carotid artery dissection**	**Left ICA**
**Aetiology**	**Spontaneous**

Table 5

Variable	Finding
**Headache**	**Yes**
**Neck pain**	**Yes (transient unilateral)**

Variable	Finding
Blurred vision	Yes
Dysarthria	Yes
Dysphagia	Yes
Tongue deviation	Yes
Tongue weakness/paralysis	Yes
Tongue atrophy	Yes
Isolated CN XII palsy	Yes
MRI	Yes
MRA	Yes
CTA	Yes
CT	Yes
Intramural hematoma	Yes
Luminal narrowing	≈50%

Table 6

Variable	Finding
Antiplatelet therapy	Aspirin
Additional therapy	NR
Clinical outcome	Complete recovery
Follow-up	3 years

Patient 3

Table 4

Variable	Finding
Age / Sex	38 years / Male
Internal carotid artery dissection	Left ICA
Aetiology	Spontaneous

Table 5

Variable	Finding
Headache	Yes

Variable	Finding
Neck pain	Yes (transient unilateral)
Blurred vision	Yes
Dysarthria	Yes
Dysphagia	Yes
Tongue deviation	Yes
Tongue weakness/paralysis	Yes
Tongue atrophy	Yes
Isolated CN XII palsy	Yes
MRI	Yes
MRA	Yes
CTA	Yes
CT	Yes
Intramural hematoma	Yes
Luminal narrowing	≈75% with ICA occlusion

Table 6

Variable	Finding
Antiplatelet therapy	Aspirin
Additional therapy	NR
Clinical outcome	Residual mild tongue deviation
Follow-up	2 years

Patient 4

Table 4

Variable	Finding
Age / Sex	47 years / Male
Internal carotid artery dissection	Left ICA
Aetiology	Trauma (bicycle accident)

Table 5

Variable	Finding

Variable	Finding
Headache	Yes
Neck pain	Yes (transient unilateral)
Blurred vision	Yes
Dysarthria	Yes
Dysphagia	Yes
Tongue deviation	Yes
Tongue weakness/paralysis	Yes
Tongue atrophy	Yes
Isolated CN XII palsy	Yes
MRI	Yes
MRA	Yes
CTA	NR
CT	NR
Intramural hematoma	Yes
Luminal narrowing	Yes

Table 6

Variable	Finding
Antiplatelet therapy	Aspirin
Additional therapy	NR
Clinical outcome	Persistent mild tongue deviation and atrophy
Follow-up	2 years

Note: Variables explicitly described by the authors as common to all four patients (e.g., tongue weakness, tongue deviation, tongue atrophy, dysphagia, dysarthria, and characteristic imaging findings) were recorded for each patient. Patient-specific variables not individually reported were recorded as **NR (not reported)**.

Jurkiewicz et al., 2019 — sintesi dei dati da integrare

Imaging comment: CTA and MRI/MRA showed typical features of extracranial internal carotid artery dissection, including **crescent-shaped intramural hematoma**, **marked luminal narrowing** (>80% in Patient 1), and **arterial wall expansion** adjacent to the hypoglossal canal. Imaging also demonstrated signs of hypoglossal denervation, such as **hemitongue asymmetry**, **bulbous protrusion into the oropharynx**, and, in chronic cases, **fatty replacement and atrophy** of the affected tongue. These findings emphasize the diagnostic value of combining vascular imaging with dedicated assessment of tongue denervation.

Table 1

Dati da aggiungere:

✓ 4 pazienti

✓ 3 uomini

✓ 1 donna

✓ 1 traumatico

✓ 3 spontanei

✓ disartria

✓ disfagia

✓ tongue atrophy

✓ tongue deviation

Table 2

Elementi radiologici da aggiungere:

✓ MRI

✓ MRA

✓ CTA

✓ intramural hematoma

✓ luminal stenosis

✓ mural expansion

✓ tongue denervation

✓ fatty replacement

✓ hypoglossal canal relationship

Nel complesso, questo studio apporta una quantità rilevante di dati clinici e radiologici.

Table 3

Dati terapeutici e di outcome da aggiungere:

✓ antiplatelet therapy

✓ miglioramento

✓ complete recovery

✓ residual deficit

✓ follow-up fino a 3 anni

Marin et al., 2009

Title: *Hypoglossal nerve palsy as the sole manifestation of spontaneous internal carotid artery dissection*

Authors: Luiz Fabiano Marin, Denis Bernardi Bichuetti, André Carvalho Felício, William Adolfo Celso dos Santos, Fabrício Ferreira de Oliveira, Márcia Elisabete Morita, Wagner Mauad Avelar, Pedro Braga-Neto, Elaine Cristina dos Santos Lima, Ricardo Nitrini

Journal: *Arquivos de Neuro-Psiquiatria*

Year: 2009

Study design: Case report

Table 4. Demographic and Etiopathogenetic Characteristics

Variable	Finding
Age / Sex	**52 years / Male**
Smoking	NR
Hypertension	No
Connective tissue disorder / genetic disease	No
Other vascular risk factors	None reported
Trauma	No
Minor cervical mechanical trigger	No
Recent infection	No
Dental procedure	No
Chiropractic manipulation	No
Vigorous cough / Valsalva	NR
Other predisposing factor	**Spontaneous ICAD**

Table 5. Clinical and Imaging Characteristics

Variable	Finding
Internal carotid artery dissection	**Yes (Left ICA)**
Side of ICA dissection	**Left**
Time from symptom onset to diagnosis	**3 hours**
Headache	**Yes (severe)**
Neck pain	No
Mandibular pain	**Yes (left jaw angle pain)**
Facial pain	**Yes**
Dysarthria	No
Dysphagia	No

Variable	Finding
Tongue deviation	**Yes (left)**
Tongue weakness	**Yes**
Tongue atrophy	No
Tongue swelling / pseudohypertrophy	No
Fasciculations	NR
Isolated CN XII palsy	**Yes**
Other cranial nerve involvement	No
Collet–Sicard syndrome	No
Horner syndrome	No
Stroke / cerebral ischemia	No
Initial misdiagnosis	No
First specialty consulted	Emergency Department
MRI	**Yes**
MRA	**Yes**
CTA	No
CT	**Yes (normal)**
Doppler ultrasound	No
Duplex ultrasound	No
DSA	**Yes (4-vessel cerebral angiography)**
Intramural hematoma	**Yes**
Double lumen	NR
Intimal tear	NR
Luminal stenosis	**Yes**
ICA occlusion	No
String sign	NR
External arterial enlargement	NR
Pseudoaneurysm / dissecting aneurysm	**Yes (cavernous ICA aneurysm)**
Direct CN XII compression demonstrated	No
Mass effect on CN XII	Suggested

Table 6. Treatment and Outcome Characteristics

Variable	Finding
Conservative treatment	**Yes**
Antiplatelet therapy	No
Anticoagulation	**Yes (heparin followed by warfarin)**
Dual antiplatelet therapy	No
Endovascular treatment	No
Surgical treatment	No
Treatment duration	NR
Clinical outcome	**Marked improvement of tongue movement**
Follow-up duration	**30 days**
Imaging follow-up	No
Complete radiological resolution	NR
Partial radiological improvement	NR
Persistent vascular abnormality	NR
Recurrence	No reported

Imaging comment

Brain CT and conventional MRI were initially normal. Diagnosis was established by **MR angiography and four-vessel digital subtraction angiography**, demonstrating a **left internal carotid artery dissection** extending from approximately **4 cm distal to the carotid bulb to the cavernous segment**, with an associated **cavernous ICA aneurysm**, illustrating the characteristic vascular appearance of spontaneous ICAD.

Table 1

aggiunge

✓ uomo

✓ 52 anni

✓ headache

✓ mandibular pain

✓ facial pain

✓ isolated XII

✓ nessun Horner

✓ nessun ictus

Table 2

aggiunge

✓ MRI

✓ MRA

✓ DSA

✓ intramural hematoma

✓ luminal stenosis

✓ pseudoaneurysm

✓ mass effect suggested

✓ no direct compression demonstrated

Table 3

aggiunge

✓ anticoagulazione

✓ recupero neurologico marcato

✓ follow-up 30 giorni

Torbus-Paluszczak et al., 2018

Title: *Hypoglossal nerve palsy in the course of dissection of the internal carotid arteries – Case reports*

Authors: Magdalena Torbus-Paluszczak, Sebastian Mucha, Maciej Wawrzyńczyk, Krystyna Pierzchała, Wojciech Bartman, Monika Adamczyk-Sowa

Journal: *Neurologia i Neurochirurgia Polska*

Year: 2018

Study design: Case series (2 patients)

Patient 1

Table 4. Demographic and Etiopathogenetic Characteristics

**Variable**	**Finding**
Age / Sex	**50 years / Male**
Smoking	NR
Hypertension	No
Connective tissue disorder / genetic disease	No
Other vascular risk factors	**Mixed dyslipidemia**
Trauma	No

Variable	Finding
Minor cervical mechanical trigger	No
Recent infection	No
Dental procedure	No
Chiropractic manipulation	No
Vigorous cough / Valsalva	No
Other predisposing factor	**Spontaneous ICAD**

Table 5. Clinical and Imaging Characteristics

Variable	Finding
Internal carotid artery dissection	**Yes (Right ICA)**
Side of ICA dissection	**Right**
Time from symptom onset to diagnosis	**4 weeks**
Headache	No
Neck pain	No
Mandibular pain	**Yes (submandibular pain)**
Facial pain	**Yes (right ear pain)**
Dysarthria	**Yes**
Dysphagia	**Yes**
Tongue deviation	**Yes (right)**
Tongue weakness	**Yes**
Tongue atrophy	**Slight**
Tongue swelling / pseudohypertrophy	No
Fasciculations	NR
Isolated CN XII palsy	**No**
Other cranial nerve involvement	No
Collet–Sicard syndrome	No
Horner syndrome	No
Stroke / cerebral ischemia	No
Initial misdiagnosis	**Yes (submandibular gland inflammation)**
First specialty consulted	**Otolaryngology (ENT)**

Variable	Finding
MRI	No
MRA	No
CTA	**Yes**
CT	**Yes**
Doppler ultrasound	**Yes**
Duplex ultrasound	**Yes**
DSA	No
Intramural hematoma	**Yes (thrombus)**
Double lumen	NR
Intimal tear	NR
Luminal stenosis	**Occlusion of RICA**
ICA occlusion	**Yes**
String sign	NR
External arterial enlargement	**Yes**
Pseudoaneurysm / dissecting aneurysm	No
Direct CN XII compression demonstrated	**Yes (reported by radiologist)**
Mass effect on CN XII	**Yes**

Table 6. Treatment and Outcome Characteristics

Variable	Finding
Conservative treatment	**Yes**
Antiplatelet therapy	No
Anticoagulation	**Unfractionated heparin → warfarin**
Dual antiplatelet therapy	No
Endovascular treatment	No
Surgical treatment	No
Treatment duration	NR
Clinical outcome	**Gradual neurological improvement**
Follow-up duration	**2 months**
Imaging follow-up	**CTA**

Variable	Finding
Complete radiological resolution	No
Partial radiological improvement	No
Persistent vascular abnormality	**Persistent RICA occlusion**
Recurrence	No reported

Imaging comment: Duplex ultrasound demonstrated right ICA occlusion caused by thrombus. CTA showed **marked enlargement of the right internal carotid artery with complete luminal thrombosis**, associated with **radiological compression of the hypoglossal nerve**, providing direct anatomical evidence of the compressive mechanism.

Patient 2

Table 4. Demographic and Etiopathogenetic Characteristics

Variable	Finding
Age / Sex	**48 years / Male**
Smoking	NR
Hypertension	**Yes**
Connective tissue disorder / genetic disease	No
Other vascular risk factors	Degenerative spine disease
Trauma	**Yes (previous car accident, 7 months earlier)**
Minor cervical mechanical trigger	No
Recent infection	**Yes (tonsillitis and sinusitis)**
Dental procedure	No
Chiropractic manipulation	No
Vigorous cough / Valsalva	No
Other predisposing factor	Bilateral conjunctivitis and right keratitis

---

Table 5. Clinical and Imaging Characteristics

Variable	Finding
Internal carotid artery dissection	**Yes (Right ICA)**
Side of ICA dissection	**Right**
Time from symptom onset to diagnosis	**5 weeks**
Headache	NR
Neck pain	NR
Mandibular pain	No

**Variable**	**Finding**
Facial pain	No
Dysarthria	**Yes (rhinolalia)**
Dysphagia	**Yes**
Tongue deviation	**Yes (right)**
Tongue weakness	**Yes**
Tongue atrophy	**Yes**
Tongue swelling / pseudohypertrophy	No
Fasciculations	NR
Isolated CN XII palsy	**No**
Other cranial nerve involvement	**CN IX involvement (uvular deviation, absent palatal/pharyngeal reflexes)**
Collet–Sicard syndrome	**No**
Horner syndrome	No
Stroke / cerebral ischemia	No
Initial misdiagnosis	**Yes (tonsillitis/sinusitis)**
First specialty consulted	**Otolaryngology (ENT)**
MRI	**Yes**
MRA	**Yes**
CTA	**Yes**
CT	**Yes**
Doppler ultrasound	**Yes**
Duplex ultrasound	**Yes**
DSA	No
Intramural hematoma	**Yes (mural thrombus)**
Double lumen	NR
Intimal tear	NR
Luminal stenosis	NR
ICA occlusion	No
String sign	NR
External arterial enlargement	**Yes**
Pseudoaneurysm / dissecting aneurysm	No

Variable	Finding
Direct CN XII compression demonstrated	No
Mass effect on CN XII	Suggested

Table 6. Treatment and Outcome Characteristics

Variable	Finding
Conservative treatment	**Yes**
Antiplatelet therapy	No
Anticoagulation	**Low-molecular-weight heparin → warfarin**
Dual antiplatelet therapy	No
Endovascular treatment	No
Surgical treatment	No
Treatment duration	**4 months of warfarin**
Clinical outcome	**Marked neurological recovery**
Follow-up duration	**7 months**
Imaging follow-up	**CTA + Duplex ultrasound**
Complete radiological resolution	**Yes**
Partial radiological improvement	NR
Persistent vascular abnormality	No
Recurrence	No reported

Imaging comment: Initial ultrasound was nondiagnostic, whereas CT angiography and MR angiography identified a **right ICA mural thrombus** consistent with dissection. Follow-up CTA performed four months later demonstrated **complete resolution of the vascular lesion**, paralleling the marked clinical recovery

Table 1

aggiunge

✓ 2 uomini

✓ 1 iperteso

✓ 1 dislipidemia

✓ trauma remoto

✓ infezione

✓ disartria

✓ disfagia

✓ un caso con coinvolgimento del XII

✓ nessun Horner

Table 2

aggiunge

✓ CTA

✓ MRI

✓ MRA

✓ Duplex

✓ Doppler

✓ ematoma

✓ trombo murale

✓ occlusione

✓ compressione dimostrata (1 caso)

✓ mass effect suggerito (1 caso)

✓ follow-up radiologico

Table 3

aggiunge

✓ eparina

✓ warfarin

✓ recupero neurologico

✓ risoluzione radiologica completa

✓ occlusione persistente

____-

Ursekar et al., 2000

Title: *Hypoglossal Nerve Palsy Due to Spontaneous Dissection of the Internal Carotid Artery*

Authors: M. A. Ursekar, B. S. Singhal, B. L. Konin

Journal: *Clinical Radiology*

Year: 2000

Study design: Case report

Table 4. Demographic and Etiopathogenetic Characteristics

**Variable**	**Finding**
Age / Sex	**37 years / Female**
Smoking	NR

Variable	Finding
Hypertension	**Mild hypertension**
Connective tissue disorder / genetic disease	No
Other vascular risk factors	Previous transient ischemic attack
Trauma	No
Minor cervical mechanical trigger	No
Recent infection	No
Dental procedure	No
Chiropractic manipulation	No
Vigorous cough / Valsalva	No
Other predisposing factor	**Spontaneous ICAD**

Table 5. Clinical and Imaging Characteristics

Variable	Finding
Internal carotid artery dissection	**Yes (Left ICA)**
Side of ICA dissection	**Left**
Time from symptom onset to diagnosis	**10 days**
Headache	**Yes**
Neck pain	No
Mandibular pain	**Yes (pain near the left jaw angle)**
Facial pain	No
Dysarthria	**Yes**
Dysphagia	No
Tongue deviation	**Yes (left)**
Tongue weakness	**Yes**
Tongue atrophy	No
Tongue swelling / pseudohypertrophy	No
Fasciculations	NR
Isolated CN XII palsy	**Yes**
Other cranial nerve involvement	No
Collet–Sicard syndrome	No

Variable	Finding
Horner syndrome	No
Stroke / cerebral ischemia	**Yes (previous left hemispheric TIA with small ischemic lesions on MRI)**
Initial misdiagnosis	No
First specialty consulted	NR
MRI	**Yes**
MRA	**Yes**
CTA	No
CT	No
Doppler ultrasound	No
Duplex ultrasound	No
DSA	**Yes**
Intramural hematoma	**Yes**
Double lumen	No
Intimal tear	NR
Luminal stenosis	**Yes (tapering stenosis)**
ICA occlusion	No
String sign	**Yes (long tapering stenosis)**
External arterial enlargement	**Yes (pseudoaneurysm)**
Pseudoaneurysm / dissecting aneurysm	**Yes (partially thrombosed pseudoaneurysm at the skull base)**
Direct CN XII compression demonstrated	**Suggested**
Mass effect on CN XII	**Yes**

Table 6. Treatment and Outcome Characteristics

Variable	Finding
Conservative treatment	**Yes**
Antiplatelet therapy	No
Anticoagulation	**Heparin followed by oral anticoagulation**
Dual antiplatelet therapy	No
Endovascular treatment	No
Surgical treatment	No

Variable	Finding
Treatment duration	**6 weeks**
Clinical outcome	**Marked improvement of tongue movement**
Follow-up duration	**6 weeks**
Imaging follow-up	No
Complete radiological resolution	NR
Partial radiological improvement	NR
Persistent vascular abnormality	NR
Recurrence	No reported

Imaging comment

MRI demonstrated the **typical appearance of extracranial internal carotid artery dissection**, with **marked luminal narrowing caused by a partially thrombosed pseudoaneurysm at the skull base**. MR angiography showed a **long tapering stenosis beginning 2–3 cm distal to the carotid bifurcation with distal luminal reconstitution**, while DSA confirmed the diagnosis. The **pseudoaneurysm at the skull base** was considered the most likely cause of **direct hypoglossal nerve compression**, explaining the isolated XII cranial nerve

Table 1

- Donna (raro nella casistica)
- 37 anni
- Ipertensione lieve
- TIA/ischemia
- Cefalea
- Dolore mandibolare
- Disartria
- Paralisi isolata del XII

Table 2

- MRI
- MRA
- DSA
- Ematoma intramurale
- String sign
- Stenosi
- Pseudoaneurisma

- Espansione arteriosa
- Mass effect
- Nessuna dimostrazione diretta del XII

Questo caso sarà uno dei pochi che contribuiranno alla frequenza dello **string sign**, una caratteristica radiologica classica ma non frequentemente descritta nei case report.

Table 3

- Anticoagulazione (eparina → anticoagulante orale)
- Recupero neurologico marcato
- Follow-up a 6 settimane
- Nessuna recidiva riportata

Chen et al., 2019

Title: *Isolated hypoglossal nerve palsy from internal carotid artery dissection related to PKD-1 gene mutation*

Authors: Zhaoyao Chen, Jun Yuan, Hui Li, Cuiping Yuan, Kailin Yin, Sen Liang, Pengfei Li, Minghua Wu

Journal: *BMC Neurology*

Year: 2019

Study design: Case report

Table 4. Demographic and Etiopathogenetic Characteristics

Variable	Finding
Age / Sex	**42 years / Male**
Internal carotid artery dissection	**Right ICA**
Smoking	NR
Hypertension	No
Connective tissue disorder	**PKD1 gene mutation (heterozygous)**
Other vascular risk factors	Mild obesity
Trauma	No
Minor cervical mechanical trigger	**Possible prolonged neck flexion while sleeping / desk nap**
Recent infection	No
Dental procedure	No
Chiropractic manipulation	No

Variable	Finding
Vigorous cough / Valsalva	No
Other predisposing factor	**Multiple small liver cysts; suspected underlying connective tissue vulnerability**

Table 5. Clinical and Imaging Characteristics

Variable	Finding
Side of ICA dissection	**Right**
Time from symptom onset to diagnosis	**2 days**
Headache	**Yes (slight)**
Neck pain	**Yes (right)**
Mandibular pain	No
Facial pain	No
Dysarthria	**Yes**
Dysphagia	No
Tongue deviation	**Yes (right)**
Tongue weakness	**Yes**
Tongue atrophy	No
Tongue swelling / pseudohypertrophy	No
Fasciculations	NR
Isolated CN XII palsy	**Yes**
Other cranial nerve involvement	No
Collet–Sicard syndrome	No
Horner syndrome	No
Stroke / cerebral ischemia	No
Initial misdiagnosis	**Yes (initial CT and MRI were normal)**
First specialty consulted	Clinic / Emergency Department
MRI	**Yes (high-resolution MRI)**
MRA	**Yes**
CTA	**Yes**
CT	**Yes (normal)**
Doppler ultrasound	No

Variable	Finding
Duplex ultrasound	No
DSA	**Yes**
Intramural hematoma	**Yes**
Double lumen	**Yes**
Intimal tear	**Yes**
Luminal stenosis	**Yes**
ICA occlusion	No
String sign	NR
External arterial enlargement	**Yes**
Pseudoaneurysm / dissecting aneurysm	No
Direct CN XII compression demonstrated	**Yes**
Mass effect on CN XII	**Yes**

Table 6. Treatment and Outcome Characteristics

Variable	Finding
Conservative treatment	**Yes**
Antiplatelet therapy	**Clopidogrel**
Anticoagulation	No
Dual antiplatelet therapy	No
Endovascular treatment	No
Surgical treatment	No
Treatment duration	NR
Clinical outcome	**Complete recovery of hypoglossal palsy**
Follow-up duration	**3 months (clinical), 6 months (CTA)**
Imaging follow-up	**CTA**
Complete radiological resolution	No
Partial radiological improvement	**Yes (marked lumen recovery with mild residual stenosis)**
Persistent vascular abnormality	**Mild residual stenosis**
Recurrence	No

Imaging comment

High-resolution MRI was the key diagnostic examination. While CTA and DSA demonstrated **segmental stenosis of the right internal carotid artery**, HRMRI additionally identified the **intramural hematoma**, **double lumen**, **intimal tear**, and **arterial wall enlargement**, allowing definitive diagnosis of ICAD. T1 fat-suppressed images showed the **hypoglossal nerve immediately adjacent to the enlarged dissected ICA**, together with **compression of the internal jugular vein** and enlargement of the structures within the hypoglossal canal, strongly supporting a **compressive mechanism** of isolated XII cranial nerve palsy. Six-month CTA demonstrated marked vascular remodeling with only mild residual stenosis.

Clinical comment

This case is particularly noteworthy because it is the first to associate **isolated hypoglossal nerve palsy due to ICAD** with a **heterozygous PKD1 gene mutation**, suggesting that an underlying genetically determined arterial wall fragility may have contributed to spontaneous dissection in an otherwise healthy young patient.

Nota metodologica: questo articolo merita una decisione preventiva nel protocollo. Se mantieni il criterio di esclusione per **qualsiasi** paziente che sviluppi una dissezione vertebrale durante il follow-up, allora dovrà essere escluso. Se invece il criterio riguarda la **presentazione clinica iniziale**, questo è uno dei case report più completi della letteratura e sarebbe opportuno mantenerlo nell'analisi.

Table 1

aggiunge

- PKD1 mutation
- obesità lieve
- trigger cervicale minore
- headache
- neck pain
- disartria

Table 2

aggiunge

- HRMRI
- CTA
- DSA
- ematoma intramurale
- double lumen
- intimal tear
- compressione diretta del XII
- compressione della vena giugulare
- rimodellamento vascolare

Questo articolo farà aumentare diverse frequenze e sarà probabilmente **il caso di riferimento** nella Discussione quando parlerai del ruolo dell'HRMRI.

Table 3

aggiunge

- clopidogrel

- recupero completo
- follow-up a 6 mesi
- miglioramento radiologico parziale
- stenosi residua lieve

____-

Fujii et al., 2014

Title: Isolated Unilateral Hypoglossal Nerve Paralysis Caused by Internal Carotid Artery Dissection

Authors: Hiroki Fujii, Toshiho Ohtsuki, Ikuko Takeda, Naohisa Hosomi, Masayasu Matsumoto

Journal: Journal of Stroke and Cerebrovascular Diseases

Year: 2014

Study design: Case report

DOI: 10.1016/j.jstrokecerebrovasdis.2014.03.004

Table 4. Demographic and Etiopathogenetic Characteristics

Variable	Finding
Age / Sex	42 years / Male
Smoking	NR
Hypertension	NR
Connective tissue/genetic disorder	NR
Other vascular risk factors	None reported; previously healthy
Trauma	No major trauma reported
Minor cervical mechanical trigger	Yes
Dental procedure	Periodontal treatment in the supine position
Interval from procedure to symptoms	4 days
Recent infection	NR
Chiropractic manipulation	No
Vigorous cough / Valsalva	No
Other predisposing factor	None reported

Table 5. Clinical and Imaging Characteristics

Variable	Finding
Internal carotid artery dissection	Yes
Side of ICA dissection	Left
Time from symptom onset to diagnosis	NR

Retro-orbital/occipital pain	Yes
Neck pain	Yes, left-sided
Mandibular pain	No
Facial pain	No
Dysarthria	Yes, subjective difficulty speaking
Dysphagia	Subjective difficulty swallowing; deglutition objectively preserved
Tongue deviation	Yes, left
Tongue weakness	Yes
Tongue atrophy	Yes, appeared after 1 week
Tongue swelling/pseudohypertrophy	No
Fasciculations	NR
Isolated CN XII palsy	Yes
Other cranial nerve involvement	No
Collet–Sicard syndrome	No
Horner syndrome	No
Stroke/cerebral ischemia	No fresh infarction
Initial misdiagnosis	NR
First specialty consulted	NR
MRI	Yes
MRA	No
CTA	Yes
CT	No non-angiographic CT reported
Doppler/Duplex ultrasound	No
DSA	No
Intramural hematoma	Yes
Double lumen	Yes
Intimal tear	NR
Luminal stenosis	NR
ICA occlusion	No
String sign	No
External arterial enlargement	Yes, dilated dissected wall
Pseudoaneurysm/dissecting aneurysm	Yes, fusiform
Direct CN XII compression demonstrated	Yes
Mass effect on CN XII	Yes, beneath the hypoglossal canal

Table 6. Treatment and Outcome Characteristics

Variable	Finding

Conservative treatment NR

Antiplatelet therapy NR

Anticoagulation NR

Dual antiplatelet therapy NR

Endovascular treatment No reported

Surgical treatment No reported

Treatment duration NR

Clinical outcome Complete clinical resolution of neck pain, tongue deviation and atrophy within a few weeks

Clinical follow-up A few weeks

Imaging follow-up CTA at 6 months

Complete radiological resolution No

Partial radiological improvement Yes

Persistent vascular abnormality Yes, residual expanded dissected arterial wall

Recurrence NR

Imaging comment

MRI showed a crescent-shaped T1- and T2-hyperintense intramural hematoma of the left ICA. CTA demonstrated the double-lumen sign and a fusiform dissecting aneurysm located beneath the foramen lacerum, approximately 6–8 cm distal to the carotid bifurcation. T2-weighted imaging depicted the dilated dissected ICA directly compressing the hypoglossal nerve beneath the hypoglossal canal, providing strong direct evidence for a compressive mechanism. Follow-up CTA at 6 months showed only partial regression of the arterial wall expansion.

____--

Shahab et al., 2001

Title: Isolated hypoglossal nerve palsy due to internal carotid artery dissection

Authors: R. Shahab, L. E. Savy, C. B. Croft, T. Hung

Journal: The Journal of Laryngology & Otology

Year: 2001

Study design: Case report

Table 4. Demographic and Etiopathogenetic Characteristics

Variable Finding

Age / Sex 46 years / Male

Smoking	Yes (≈25 cigarettes/day)
Hypertension	NR
Connective tissue/genetic disorder	No
Other vascular risk factors	Occasional alcohol consumption
Trauma	No
Minor cervical mechanical trigger	No
Recent infection	Yes (sore throat 2 weeks before)
Dental procedure	No
Chiropractic manipulation	No
Vigorous cough / Valsalva	No
Other predisposing factor	Spontaneous ICAD

Table 5. Clinical and Imaging Characteristics

Variable	Finding
Internal carotid artery dissection	Yes
Side of ICA dissection	Left
Time from symptom onset to diagnosis	Approximately 2 weeks
Headache	No
Neck pain	No
Mandibular pain	No
Facial pain	No
Dysarthria	Yes
Dysphagia	Yes
Tongue deviation	Yes (left)
Tongue weakness	Yes
Tongue atrophy	No
Tongue swelling / pseudohypertrophy	Yes (apparent tongue-base pseudomass)
Fasciculations	Yes
Isolated CN XII palsy	Yes
Other cranial nerve involvement	No
Collet–Sicard syndrome	No
Horner syndrome	No
Stroke / cerebral ischemia	No
Initial misdiagnosis	Yes (suspected tongue-base lesion leading to panendoscopy and biopsies)
First specialty consulted	ENT
MRI	Yes
MRA	No

CTA	No
CT	Yes (contrast-enhanced CT)
Doppler ultrasound	No
Duplex ultrasound	No
DSA	No
Intramural hematoma	Yes
Double lumen	No
Intimal tear	NR
Luminal stenosis	Mild luminal narrowing
ICA occlusion	No
String sign	No
External arterial enlargement	Yes
Pseudoaneurysm / dissecting aneurysm	Yes (dissecting aneurysm/intramural hematoma)
Direct CN XII compression demonstrated	Yes
Mass effect on CN XII	Yes

Table 6. Treatment and Outcome Characteristics

Variable	Finding
Conservative treatment	Yes
Antiplatelet therapy	Aspirin 75 mg/day
Anticoagulation	No
Dual antiplatelet therapy	No
Endovascular treatment	No
Surgical treatment	No
Treatment duration	NR
Clinical outcome	Scheduled clinical follow-up (outcome not reported)
Follow-up duration	6 months planned
Imaging follow-up	NR
Complete radiological resolution	NR
Partial radiological improvement	NR
Persistent vascular abnormality	NR
Recurrence	NR
Imaging comment	

MRI demonstrated the classic crescent-shaped intramural hematoma surrounding the patent lumen of the left internal carotid artery, extending from the C2 level to the proximal carotid canal. Contrast-enhanced CT confirmed a circumferential intramural hematoma with mild luminal narrowing, consistent with spontaneous ICAD. The dissected arterial wall occupied the carotid space adjacent to the expected course of the hypoglossal nerve, supporting a compressive mechanism of isolated XII nerve palsy. A particularly instructive

feature was the apparent enlargement ("pseudomass") of the ipsilateral tongue base, caused by denervation rather than neoplasia, emphasizing the importance of careful neuroradiological review before invasive diagnostic procedures.

-____-

Boukobza et al., 1998

Title: Internal Carotid Artery Dissection Causes Hypoglossal Nerve Palsy: CT, MRI, and Angiographic Findings

Authors: M. Boukobza, G. Ast, D. Reizine, J. J. Merland

Journal: Journal of Neuroimaging

Year: 1998

Study design: Case report

Table 4. Demographic and Etiopathogenetic Characteristics

Variable	Finding
Age / Sex	38 years / Female
Smoking	NR
Hypertension	No (BP 140/80 mmHg at presentation)
Connective tissue/genetic disorder	NR
Other vascular risk factors	None reported
Trauma	No
Minor cervical mechanical trigger	Yes (yawning)
Dental procedure	No
Recent infection	No
Chiropractic manipulation	No
Vigorous cough / Valsalva	No
Other predisposing factor	None reported

Table 5. Clinical and Imaging Characteristics

Variable	Finding
Internal carotid artery dissection	Yes
Side of ICA dissection	Left
Time from symptom onset to diagnosis	4 days

Headache	No
Neck pain	Yes (left cervical pain)
Facial pain	Yes (left hemifacial pain)
Mandibular pain	No
Dysarthria	Yes (speech disturbance)
Dysphagia	Yes
Tongue deviation	Yes (left)
Tongue weakness	Yes
Tongue atrophy	No
Tongue swelling / pseudohypertrophy	No
Fasciculations	NR
Isolated CN XII palsy	Yes
Other cranial nerve involvement	No
Collet–Sicard syndrome	No
Horner syndrome	No
Stroke / cerebral ischemia	No
Initial misdiagnosis	Yes (initial head CT, neck CT and MRI interpreted as normal)
MRI	Yes
MRA	No
CTA	No
CT	Yes
Doppler ultrasound	No
Duplex ultrasound	No
DSA	Yes
Intramural hematoma	Yes
Double lumen	No
Intimal tear	NR
Luminal stenosis	Yes
ICA occlusion	No
String sign	Yes
External arterial enlargement	Yes
Pseudoaneurysm / dissecting aneurysm	No
Direct CN XII compression demonstrated	Suggested
Mass effect on CN XII	Yes

Table 6. Treatment and Outcome Characteristics

Variable	Finding

Conservative treatment Yes

Anticoagulation Long-term anticoagulation

Antiplatelet therapy No

Dual antiplatelet therapy No

Endovascular treatment No

Surgical treatment No

Treatment duration NR

Clinical outcome Marked clinical improvement

Clinical follow-up 3 months

Imaging follow-up MRI + Digital angiography

Complete radiological resolution Yes (resolution of stenosis and mural thrombus)

Partial radiological improvement No

Persistent vascular abnormality No

Recurrence No

Imaging comment

MRI demonstrated a T1- and T2-hyperintense intramural hematoma extending from the C3 level to the skull base, producing marked external enlargement of the left internal carotid artery with luminal narrowing. Selective carotid angiography confirmed the diagnosis by demonstrating the classic "string sign". Unlike several later reports, no pseudoaneurysm was identified. Follow-up MRI and angiography at 3 months showed complete resolution of the mural hematoma and restoration of the normal arterial lumen, documenting both anatomical healing and clinical recovery.

Okunomiya et al., 2012

Title: Teaching NeuroImages: Isolated hypoglossal nerve palsy due to internal carotid artery dissection

Authors: Taro Okunomiya, Takashi Kageyama, Toshihiko Suenaga

Journal: Neurology

Year: 2012

Study design: Case report (Teaching NeuroImages)

Table 4. Demographic and Etiopathogenetic Characteristics

Variable Finding

Age / Sex 57 years / Male

Smoking	NR
Hypertension	NR
Connective tissue/genetic disorder	NR
Other vascular risk factors	NR
Trauma	No
Minor cervical mechanical trigger	NR
Dental procedure	No
Recent infection	NR
Chiropractic manipulation	No
Vigorous cough / Valsalva	No
Other predisposing factor	None reported

Table 5. Clinical and Imaging Characteristics

Variable	Finding
Internal carotid artery dissection	Yes
Side of ICA dissection	Left
Time from symptom onset to diagnosis	4 days
Headache	Yes (left-sided)
Neck pain	NR
Facial pain	No
Mandibular pain	No
Dysarthria	Yes
Dysphagia	NR
Tongue deviation	Yes (left)
Tongue weakness	Yes
Tongue atrophy	No
Tongue swelling / pseudohypertrophy	No
Fasciculations	NR
Isolated CN XII palsy	Yes
Other cranial nerve involvement	No
Collet–Sicard syndrome	No
Horner syndrome	No
Stroke / cerebral ischemia	No
Initial misdiagnosis	NR
MRI	Yes
MRA	NR
CTA	No

CT	No
Doppler ultrasound	No
Duplex ultrasound	No
DSA	No
Intramural hematoma	Yes
Double lumen	NR
Intimal tear	NR
Luminal stenosis	Yes
ICA occlusion	No
String sign	NR
External arterial enlargement	Yes
Pseudoaneurysm / dissecting aneurysm	NR
Direct CN XII compression demonstrated	Yes (anatomical juxtaposition)
Mass effect on CN XII	Suggested

Table 6. Treatment and Outcome Characteristics

Variable	Finding
Conservative treatment	Yes
Antiplatelet therapy	Aspirin
Anticoagulation	No
Dual antiplatelet therapy	No
Endovascular treatment	No
Surgical treatment	No
Treatment duration	4 weeks
Clinical outcome	Complete resolution of tongue deviation
Clinical follow-up	4 weeks
Imaging follow-up	NR
Complete radiological resolution	NR
Partial radiological improvement	NR
Persistent vascular abnormality	NR
Recurrence	NR

Imaging comment

T1-weighted MRI demonstrated the typical intramural hematoma with luminal narrowing of the left internal carotid artery, consistent with arterial dissection. A notable feature of this report is the use of true fast imaging with steady-state precession (true FISP), which directly visualized the close anatomical relationship between the dissected internal carotid artery and the hypoglossal nerve. This high-resolution sequence provided compelling anatomical support for the compressive mechanism of isolated hypoglossal nerve palsy, complementing the conventional MRI findings.

Clinical comment

Although brief, this Teaching NeuroImages report is particularly valuable because it provides direct anatomical visualization of the hypoglossal nerve adjacent to the dissected ICA, reinforcing the hypothesis that extrinsic compression by the expanded arterial wall, rather than ischemic injury of the nerve, is the principal mechanism underlying isolated hypoglossal nerve palsy in ICAD.

____-

Riancho et al., 2013

Title: Unilateral Isolated Hypoglossal Nerve Palsy Associated with Internal Carotid Artery Dissection

Authors: Javier Riancho, Jon Infante, Jose Ignacio Mateo, Jose Berciano, Leire Agea

Journal: Journal of Neurology, Neurosurgery & Psychiatry

Year: 2013

Study design: Case report

Table 4. Demographic and Etiopathogenetic Characteristics

Variable	Finding
Age / Sex	54 years / Male
Smoking	No
Hypertension	Mild hypertension
Connective tissue/genetic disorder	No
Other vascular risk factors	None reported
Trauma	No
Minor cervical mechanical trigger	No
Dental procedure	No
Recent infection	No
Chiropractic manipulation	No
Vigorous cough / Valsalva	No
Other predisposing factor	Spontaneous ICAD

Table 5. Clinical and Imaging Characteristics

Variable	Finding
Internal carotid artery dissection	Yes

Side of ICA dissection	Left
Time from symptom onset to diagnosis	2 weeks
Headache	No
Neck pain	No
Facial pain	No
Mandibular pain	No
Dysarthria	Yes (mild)
Dysphagia	Yes (mild)
Tongue deviation	Yes (left)
Tongue weakness	Yes
Tongue atrophy	No
Tongue swelling / pseudohypertrophy	No
Fasciculations	NR
Isolated CN XII palsy	Yes
Other cranial nerve involvement	No
Collet–Sicard syndrome	No
Horner syndrome	No
Stroke / cerebral ischemia	No
Initial misdiagnosis	No
First specialty consulted	NR
MRI	Yes
MRA	Yes (angiographic MRI)
CTA	No
CT	No
Doppler ultrasound	No
Duplex ultrasound	No
DSA	No
Intramural hematoma	Yes
Double lumen	No
Intimal tear	NR
Luminal stenosis	Yes (≈30%)
ICA occlusion	No
String sign	No
External arterial enlargement	NR
Pseudoaneurysm / dissecting aneurysm	No
Direct CN XII compression demonstrated	No

Mass effect on CN XII	Not demonstrated

Table 6. Treatment and Outcome Characteristics

Variable	Finding
Conservative treatment	Yes
Antiplatelet therapy	Yes
Anticoagulation	No
Dual antiplatelet therapy	No
Endovascular treatment	No
Surgical treatment	No
Treatment duration	NR
Clinical outcome	Marked improvement of dysarthria and tongue mobility
Clinical follow-up	3 weeks
Imaging follow-up	Recommended, not reported
Complete radiological resolution	NR
Partial radiological improvement	NR
Persistent vascular abnormality	NR
Recurrence	No reported

Imaging comment

Brain MRI with angiographic sequences demonstrated a 1.4-cm dissection of the distal left internal carotid artery, producing approximately 30% luminal narrowing associated with an intramural hematoma. Unlike several previous reports, no signal abnormality of the hypoglossal nerve and no direct radiological evidence of nerve compression were identified. MRI angiographic reconstruction confirmed the diagnosis of distal extracranial ICAD without cerebral ischemia.

Hafkamp et al., 2004

Title: Unilateral spontaneous dissection of the internal carotid artery presenting as hypoglossal nerve palsy

Authors: Harriët C. Hafkamp, Ann van der Goten, Johannes J. Manni

Journal: European Archives of Oto-Rhino-Laryngology

Year: 2004

Study design: Case report

Table 4. Demographic and Etiopathogenetic Characteristics

Variable	Finding
Age / Sex	61 years / Male
Smoking	Yes (15 cigarettes/day)
Hypertension	No
Connective tissue/genetic disorder	No
Other vascular risk factors	Occasional alcohol consumption
Trauma	No
Minor cervical mechanical trigger	No
Dental procedure	No
Recent infection	No
Chiropractic manipulation	No
Vigorous cough / Valsalva	No
Other predisposing factor	Spontaneous ICAD

Table 5. Clinical and Imaging Characteristics

Variable	Finding
Internal carotid artery dissection	Yes
Side of ICA dissection	Left
Time from symptom onset to diagnosis	3 months
Headache	Yes (chronic left-sided headache)
Neck pain	No
Facial pain	No
Mandibular pain	No
Dysarthria	Yes (mild)
Dysphagia	Yes
Tongue deviation	Yes (left)
Tongue weakness	Yes
Tongue atrophy	Yes
Tongue swelling / pseudohypertrophy	Apparent bulky tongue base due to denervation
Fasciculations	Yes
Isolated CN XII palsy	Yes
Other cranial nerve involvement	No
Collet–Sicard syndrome	No
Horner syndrome	No
Stroke / cerebral ischemia	No
Initial misdiagnosis	Yes (suspected tongue-base mass)

First specialty consulted	ENT
MRI	Yes
MRA	No
CTA	No
CT	No
Doppler ultrasound	No
Duplex ultrasound	No
DSA	No
Intramural hematoma	Yes
Double lumen	No
Intimal tear	NR
Luminal stenosis	Minimal
ICA occlusion	No
String sign	No
External arterial enlargement	Yes
Pseudoaneurysm / dissecting aneurysm	No
Direct CN XII compression demonstrated	Suggested
Mass effect on CN XII	Yes

Table 6. Treatment and Outcome Characteristics

Variable	Finding
Conservative treatment	Yes
Antiplatelet therapy	No
Anticoagulation	No (diagnosis delayed)
Dual antiplatelet therapy	No
Endovascular treatment	No
Surgical treatment	No
Treatment duration	NR
Clinical outcome	Marked improvement of tongue function
Clinical follow-up	3 months
Imaging follow-up	NR
Complete radiological resolution	NR
Partial radiological improvement	NR
Persistent vascular abnormality	NR
Recurrence	No reported
Imaging comment	

MRI demonstrated the characteristic appearance of extracranial left internal carotid artery dissection, with a crescent-shaped T1- and T2-hyperintense intramural hematoma surrounding a minimally narrowed flow void. The arterial enlargement occurred without significant luminal compromise, consistent with a subadventitial dissection. MRI also showed an apparent enlargement of the ipsilateral tongue base caused by denervation, a finding that may mimic a neoplasm. The authors emphasize that MRI is the imaging modality of choice because it can identify mural expansion even when arterial flow remains largely preserved.

Olzowy et al., 2006

Title: Bilateral and unilateral internal carotid artery dissection causing isolated hypoglossal nerve palsy: a case report and review of the literature

Authors: B. Olzowy, S. Lorenzl, R. Guerkov

Journal: European Archives of Oto-Rhino-Laryngology

Year: 2006

Study design: Case series (2 original patients) + review della letteratura.

PATIENT 1

Table 4. Demographic and Etiopathogenetic Characteristics

Variable	Finding
Age / Sex	48 years / Male
Smoking	NR
Hypertension	NR
Connective tissue/genetic disorder	NR
Trauma	No
Recent infection	No
Minor cervical trigger	No
Dental procedure	No
Other predisposing factor	Spontaneous

Table 5. Clinical and Imaging Characteristics

Variable	Finding
Internal carotid artery dissection	Yes
Side of ICA dissection	Bilateral ⚠️
Time to diagnosis	≈2 weeks

Headache	Yes (left)
Neck pain	No
Dysarthria	Yes
Dysphagia	No
Tongue deviation	Left
Tongue atrophy	Beginning
Fasciculations	Yes
Isolated CN XII palsy	Yes
Other cranial nerves	No
Collet-Sicard syndrome	No
Horner syndrome	No
Stroke / ischemia	No
Initial misdiagnosis	Lingual tonsillitis
MRI	Yes
MRA	Yes
CT	Yes
Doppler	Yes
Intramural hematoma	Yes
ICA occlusion	Left ICA occlusion
Contralateral ICA	Severe stenotic dissection
Pseudoaneurysm	No

Table 6. Treatment and Outcome Characteristics

Variable	Finding
Anticoagulation	Yes
Antiplatelet	No
Endovascular	No
Surgery	No
Clinical outcome	Stable during admission
Follow-up MRI	No cerebral ischemia

PATIENT 2

Table 4. Demographic and Etiopathogenetic Characteristics

Variable	Finding
Age / Sex	63 years / Male
Smoking	NR
Hypertension	NR
Trauma	No

Recent infection	Sore throat
Minor trigger	No
Other predisposing factor	Spontaneous

Table 5. Clinical and Imaging Characteristics

Variable	Finding
Internal carotid artery dissection	Yes
Side of ICA dissection	Right
Time to diagnosis	≈4 weeks
Headache	Yes
Neck pain	No
Dysarthria	NR
Dysphagia	Yes
Tongue deviation	Right
Tongue atrophy	Beginning
Fasciculations	NR
Isolated CN XII palsy	Yes
Other cranial nerves	No
Collet-Sicard syndrome	No
Horner syndrome	No
Stroke / ischemia	No
Initial misdiagnosis	Lingual tonsillitis
MRI	Yes
MRA	Yes
CT	Yes
Doppler	Normal
Intramural hematoma	Yes
Luminal flow	Preserved
Pseudoaneurysm	No

Table 6. Treatment and Outcome Characteristics

Variable	Finding
Anticoagulation	Yes
Antiplatelet	No
Endovascular	No
Surgery	No
Clinical outcome	Stable
Follow-up MRI	No cerebral ischemia

Imaging comment

Ferlazzo et al., 2013

Title: *Isolated hypoglossal nerve palsy due to spontaneous carotid artery dissection: a neuroimaging study*

Authors: Edoardo Ferlazzo, Sara Gasparini, Luciano Arcudi, Paolo Versace, Umberto Aguglia

Journal: *Neurological Sciences*

Year: 2013

Study design: Letter to the Editor (Case report)

Table 4. Demographic and Etiopathogenetic Characteristics

Variable	Finding
Age / Sex	**37 years / Male**
Smoking	NR
Hypertension	No
Connective tissue/genetic disorder	NR
Other vascular risk factors	None reported
Trauma	**No major trauma**
Minor cervical mechanical trigger	No
Dental procedure	No
Recent infection	No
Chiropractic manipulation	No
Vigorous cough / Valsalva	No
Other predisposing factor	**Spontaneous ICAD**

Table 5. Clinical and Imaging Characteristics

Variable	Finding
Internal carotid artery dissection	**Yes**
Side of ICA dissection	**Left**
Time from symptom onset to diagnosis	**6 days**
Headache	No
Neck pain	No
Mandibular pain	**Yes (left mandibular angle pain)**
Facial pain	No

**Variable**	**Finding**
Dysarthria	**Yes**
Dysphagia	No
Tongue deviation	**Yes (left)**
Tongue weakness	**Yes**
Tongue atrophy	No
Tongue swelling / pseudohypertrophy	No
Fasciculations	NR
Isolated CN XII palsy	**Yes**
Other cranial nerve involvement	**No**
Collet–Sicard syndrome	**No**
Horner syndrome	**No**
Stroke / cerebral ischemia	**No**
Initial misdiagnosis	No
MRI	**Yes**
MRA	**Yes (TOF-MRA)**
CTA	**Yes (brain CTA)**
CT	No (CTA only)
Doppler ultrasound	No
Duplex ultrasound	No
DSA	No
Intramural hematoma	**Yes**
Double lumen	NR
Intimal tear	NR
Luminal stenosis	**Mild**
ICA occlusion	No
String sign	No
External arterial enlargement	**Yes (expansion into parapharyngeal space)**
Pseudoaneurysm / dissecting aneurysm	No
Direct CN XII compression demonstrated	**Yes**
Mass effect on CN XII	**Yes**

Table 6. Treatment and Outcome Characteristics

Variable	Finding
Conservative treatment	**Yes**
Antiplatelet therapy	No
Anticoagulation	**Yes**
Dual antiplatelet therapy	No
Endovascular treatment	No
Surgical treatment	No
Treatment duration	NR
Clinical outcome	**Partial regression of symptoms**
Clinical follow-up	**1 month**
Imaging follow-up	NR
Complete radiological resolution	NR
Partial radiological improvement	NR
Persistent vascular abnormality	NR
Recurrence	No

Imaging comment

MRI and **time-of-flight MR angiography (TOF-MRA)** demonstrated a **left intramural hematoma with the characteristic semilunar morphology**, causing **mild luminal narrowing** but, soprattutto, **marked expansion of the arterial wall into the parapharyngeal space**. A punto di forza di questo lavoro è che le immagini mostrano direttamente come l'ematoma occupi lo spazio normalmente attraversato dai nervi cranici inferiori, rendendo **non più visibile il nervo ipoglosso** sul lato affetto. Inoltre, la DWI escludeva completamente lesioni ischemiche cerebrali, rafforzando l'ipotesi di un **meccanismo compressivo** piuttosto che ischemico.

Spitzer et al., 2001

Title: Isolated Hypoglossal Nerve Palsy Caused by Carotid Artery Dissection – The Necessity of MRI for Diagnosis

Authors: Christoph Spitzer, Michael Mull, Rudolf Töpper

Journal: Journal of Neurology

Year: 2001

Study design: Case report

Table 4. Demographic and Etiopathogenetic Characteristics

Variable	Finding
Age / Sex	39 years / Male
Smoking	NR
Hypertension	No
Connective tissue/genetic disorder	NR
Other vascular risk factors	None reported
Trauma	No
Minor cervical mechanical trigger	No
Dental procedure	No
Recent infection	No
Chiropractic manipulation	No
Vigorous cough / Valsalva	No
Other predisposing factor	Spontaneous ICAD

Table 5. Clinical and Imaging Characteristics

Variable	Finding
Internal carotid artery dissection	Yes
Side of ICA dissection	Left
Time from symptom onset to diagnosis	≈10 days
Headache	No
Neck pain	No
Mandibular pain	Yes (persistent pain at the left mandibular angle)
Facial pain	No
Dysarthria	NR
Dysphagia	No
Tongue deviation	Yes (left)
Tongue weakness	Yes
Tongue atrophy	No
Tongue swelling / pseudohypertrophy	No
Fasciculations	NR
Isolated CN XII palsy	Yes
Other cranial nerve involvement	No

Collet–Sicard syndrome	No
Horner syndrome	No
Stroke / cerebral ischemia	No
Initial misdiagnosis	Yes (CT and ultrasound were negative)
MRI	Yes
MRA	Yes
CTA	No
CT	Yes (normal)
Doppler ultrasound	Negative
Duplex ultrasound	Negative
DSA	No
Intramural hematoma	Yes
Double lumen	No
Intimal tear	NR
Luminal stenosis	No significant narrowing
ICA occlusion	No
String sign	No
External arterial enlargement	Yes (eccentric expansion)
Pseudoaneurysm / dissecting aneurysm	No
Direct CN XII compression demonstrated	Suggested
Mass effect on CN XII	Yes (presumed)

Table 6. Treatment and Outcome Characteristics

Variable	Finding
Conservative treatment	Yes
Antiplatelet therapy	No
Anticoagulation	Phenprocoumon
Dual antiplatelet therapy	No
Endovascular treatment	No
Surgical treatment	No
Treatment duration	8 months
Clinical outcome	Complete recovery of tongue movement
Clinical follow-up	8 months
Imaging follow-up	MRI
Complete radiological resolution	Yes
Partial radiological improvement	No
Persistent vascular abnormality	No

Recurrence No

Imaging comment

MRI demonstrated a 20-mm intramural hematoma extending from the distal cervical ICA into the proximal petrous segment. The lesion appeared as the classic semilunar T1- and T2-hyperintense mural hematoma, with preserved flow void and no significant luminal narrowing, indicating a predominantly subadventitial dissection. The maximal eccentric expansion was located on the dorsolateral wall, immediately adjacent to the expected course of the hypoglossal nerve, strongly supporting a compressive mechanism. Importantly, both Doppler and duplex ultrasonography were completely negative, whereas MRI established the diagnosis and documented complete radiological resolution after 8 months.

____-

Abukeshek et al., 2022

Title: Hypoglossal nerve palsy due to internal carotid artery dissection with pseudoaneurysm formation: An unusual presentation

Authors: Tawfiq Abukeshek, Pihou Gbande, Raed Hamed

Journal: Acta Radiologica Open

Year: 2022

Study design: Case report.

Table 4. Demographic and Etiopathogenetic Characteristics

Variable	Finding
Age / Sex	56 years / Male
Smoking	Heavy smoker (≈20 pack-years)
Hypertension	No
Connective tissue/genetic disorder	No
Other vascular risk factors	None reported
Trauma	No
Minor cervical mechanical trigger	No
Dental procedure	No
Recent infection	No
Chiropractic manipulation	No
Vigorous cough / Valsalva	No

Other predisposing factor	Spontaneous ICAD

Table 5. Clinical and Imaging Characteristics

Variable	Finding
Internal carotid artery dissection	Yes
Side of ICA dissection	Left
Time from symptom onset to diagnosis	≈6 weeks
Headache	No
Neck pain	No
Mandibular pain	No
Facial pain	No
Dysarthria	Yes (progressive)
Dysphagia	Yes (liquids)
Difficulty chewing	Yes
Tongue deviation	Yes (left)
Tongue weakness	Yes
Tongue atrophy	Yes (left hemitongue)
Tongue denervation (MRI)	Yes
Tongue swelling / base enlargement	Yes
Fasciculations	NR
Isolated CN XII palsy	Yes
Other cranial nerve involvement	No
Collet–Sicard syndrome	No
Horner syndrome	No
Stroke / cerebral ischemia	No
Initial misdiagnosis	No
MRI	Yes
MRA	Yes
CTA	Yes (follow-up)
CT	No
Doppler ultrasound	No
Duplex ultrasound	No
DSA	Yes
Intramural hematoma	Yes
Double lumen	NR
Intimal flap	NR
Luminal stenosis	Yes

ICA occlusion	No
External arterial enlargement	Yes
Pseudoaneurysm / dissecting aneurysm	Yes
Direct CN XII compression demonstrated	Yes
Mass effect on CN XII	Yes (adjacent to hypoglossal canal)

Table 6. Treatment and Outcome Characteristics

Variable	Finding
Conservative treatment	No
Antiplatelet therapy	NR
Anticoagulation	NR
Endovascular treatment	Yes (carotid stenting)
Surgical treatment	No
Treatment duration	NR
Clinical outcome	Complete resolution of dysarthria; partial improvement of mastication and dysphagia
Clinical follow-up	3 months
Imaging follow-up	CTA
Complete radiological resolution	Pseudoaneurysm resolved after stenting
Partial radiological improvement	Yes
Persistent vascular abnormality	Patent carotid stent
Recurrence	No
Imaging comment	

Abukeshek et al., 2022

Title: Hypoglossal nerve palsy due to internal carotid artery dissection with pseudoaneurysm formation: An unusual presentation

Authors: Tawfiq Abukeshek, Pihou Gbande, Raed Hamed

Journal: Acta Radiologica Open

Year: 2022

Study design: Case report.

Table 4. Demographic and Etiopathogenetic Characteristics

Variable	Finding
Age / Sex	56 years / Male
Smoking	Heavy smoker (≈20 pack-years)
Hypertension	No
Connective tissue/genetic disorder	No
Other vascular risk factors	None reported
Trauma	No
Minor cervical mechanical trigger	No
Dental procedure	No
Recent infection	No
Chiropractic manipulation	No
Vigorous cough / Valsalva	No
Other predisposing factor	Spontaneous ICAD

Table 5. Clinical and Imaging Characteristics

Variable	Finding
Internal carotid artery dissection	Yes
Side of ICA dissection	Left
Time from symptom onset to diagnosis	≈6 weeks
Headache	No
Neck pain	No
Mandibular pain	No
Facial pain	No
Dysarthria	Yes (progressive)
Dysphagia	Yes (liquids)
Difficulty chewing	Yes
Tongue deviation	Yes (left)
Tongue weakness	Yes
Tongue atrophy	Yes (left hemitongue)
Tongue denervation (MRI)	Yes
Tongue swelling / base enlargement	Yes
Fasciculations	NR
Isolated CN XII palsy	Yes
Other cranial nerve involvement	No
Collet–Sicard syndrome	No
Horner syndrome	No

Stroke / cerebral ischemia	No
Initial misdiagnosis	No
MRI	Yes
MRA	Yes
CTA	Yes (follow-up)
CT	No
Doppler ultrasound	No
Duplex ultrasound	No
DSA	Yes
Intramural hematoma	Yes
Double lumen	NR
Intimal flap	NR
Luminal stenosis	Yes
ICA occlusion	No
External arterial enlargement	Yes
Pseudoaneurysm / dissecting aneurysm	Yes
Direct CN XII compression demonstrated	Yes
Mass effect on CN XII	Yes (adjacent to hypoglossal canal)

Table 6. Treatment and Outcome Characteristics

Variable	Finding
Conservative treatment	No
Antiplatelet therapy	NR
Anticoagulation	NR
Endovascular treatment	Yes (carotid stenting)
Surgical treatment	No
Treatment duration	NR
Clinical outcome	Complete resolution of dysarthria; partial improvement of mastication and dysphagia
Clinical follow-up	3 months
Imaging follow-up	CTA
Complete radiological resolution	Pseudoaneurysm resolved after stenting
Partial radiological improvement	Yes
Persistent vascular abnormality	Patent carotid stent
Recurrence	No

Imaging comment

Questo è probabilmente uno degli articoli con la documentazione radiologica più completa dell'intera review. La MRI con sequenze T1 fat-saturated dimostra il classico ematoma intramurale a semiluna, associato a stenosi del lume della carotide interna sinistra e a

pseudoaneurisma in corrispondenza della base cranica, immediatamente adiacente all'emergenza del nervo ipoglosso. Le immagini STIR evidenziano inoltre i segni diretti di denervazione della lingua (iperintensità della metà sinistra della lingua, atrofia e protrusione della base linguale nell'orofaringe), fornendo una correlazione anatomico-funzionale molto convincente tra la dissezione carotidea e la neuropatia del XII nervo. L'angiografia conferma il pseudoaneurisma e consente il trattamento mediante stenting, mentre la CTA di controllo a 3 mesi documenta la completa scomparsa del pseudoaneurisma e la normale pervietà della carotide trattata.

Clinical comment

Questo caso è particolarmente rilevante perché rappresenta uno dei pochi esempi in cui la paralisi isolata del nervo ipoglosso è stata trattata con successo mediante approccio endovascolare, anziché con sola terapia medica. Inoltre, la presenza contemporanea di pseudoaneurisma, compressione diretta del nervo e segni RM di denervazione muscolare della lingua costituisce una delle più solide dimostrazioni del meccanismo compressivo proposto nella letteratura per spiegare la neuropatia del XII nervo in corso di dissezione della carotide interna

____-

Lindsay et al., 2003

Title: *Subacute Hypoglossal Nerve Paresis with Internal Carotid Artery Dissection*

Authors: Fred W. Lindsay, David Mullin, Michael A. Keefe

Journal: *The Laryngoscope*

Year: 2003

Study design: Case report.

Table 4. Demographic and Etiopathogenetic Characteristics

**Variable**	**Finding**
Age / Sex	**43 years / Male**
Smoking	**Yes (20 pack-years)**
Hypertension	No
Connective tissue/genetic disorder	NR
Other vascular risk factors	None reported
Trauma	**No**
Minor cervical mechanical trigger	No
Dental procedure	No
Recent infection	No
Chiropractic manipulation	No
Vigorous cough / Valsalva	No
Other predisposing factor	**Spontaneous ICAD**

Table 5. Clinical and Imaging Characteristics

Variable	Finding
Internal carotid artery dissection	**Yes**
Side of ICA dissection	**Left**
Time from symptom onset to diagnosis	**3 weeks**
Headache	**No**
Neck pain	No
Mandibular pain	No
Facial pain	No
Dysarthria	**Yes**
Dysphagia	**Yes (mild, liquids)**
Difficulty chewing	No
Tongue deviation	**Yes (left)**
Tongue weakness	**Yes**
Tongue atrophy	No
Tongue denervation (MRI)	NR
Tongue swelling / pseudohypertrophy	No
Fasciculations	NR
Isolated CN XII palsy	**Yes**
Other cranial nerve involvement	**No**
Collet–Sicard syndrome	**No**
Horner syndrome	**No**
Stroke / cerebral ischemia	**No**
Initial misdiagnosis	No
MRI	**Yes**
MRA	**Yes**
CTA	No
CT	No
Doppler ultrasound	NR
Duplex ultrasound	NR
DSA	**Yes**
Intramural hematoma	**Yes**

Variable	Finding
Double lumen	No
Intimal flap	NR
Luminal stenosis	**Yes ("string sign")**
ICA occlusion	No
External arterial enlargement	No
Pseudoaneurysm / dissecting aneurysm	**No**
Direct CN XII compression demonstrated	**Suggested**
Mass effect on CN XII	**Suggested**

Table 6. Treatment and Outcome Characteristics

Variable	Finding
Conservative treatment	**Yes**
Antiplatelet therapy	No
Anticoagulation	**Yes (Heparin → Warfarin)**
Dual antiplatelet therapy	No
Endovascular treatment	No
Surgical treatment	No
Treatment duration	**5 months (Warfarin)**
Clinical outcome	**Complete recovery**
Clinical follow-up	**≈7 months**
Imaging follow-up	**MRA**
Complete radiological resolution	**Yes**
Partial radiological improvement	No
Persistent vascular abnormality	No
Recurrence	No

Imaging comment

La **MRI e la MRA** identificano una dissezione della **carotide interna sinistra a livello della base cranica**, mentre l'angiografia conferma il caratteristico "**string sign**", indicativo di una dissezione **subintimale** con restringimento progressivo del lume **senza formazione di pseudoaneurisma**. A differenza di altri casi della review, l'aspetto radiologico è dominato dalla stenosi luminale piuttosto che dall'espansione aneurismatica della parete. La MRA di follow-up a 5 mesi documenta la completa risoluzione della dissezione.

CASE REPORT

Mes et al., 2018

Table 4. Patient demographics

Variable	Finding
Age	42 years
Sex	Male
Internal carotid artery dissection	Yes
Side of ICA dissection	Left
Aetiology	Spontaneous
Minor trauma	No
Other arterial dissections	No

Table 5. Clinical and imaging findings

Variable	Finding
Isolated CN XII palsy	Yes
Other cranial nerve involvement	No
Collet–Sicard syndrome	No
Horner syndrome	No
Stroke / cerebral ischemia	No
Headache	No
Neck pain	No
Mandibular pain	No
Dysarthria	Yes
Dysphagia	Yes
Pseudoaneurysm	No
Intramural hematoma	Yes
ICA stenosis	Severe
Double lumen	Not reported
Imaging modalities	MRI, CT angiography, Duplex Doppler, tongue EMG

Table 6. Treatment and outcome

Variable	Finding
Medical treatment	Aspirin 75 mg/day + Ramipril 5 mg/day
Surgery / Endovascular treatment	No

Variable	Finding
Clinical outcome	Complete recovery
Imaging follow-up	Near-complete recanalization of the left ICA after 15 weeks
Follow-up duration	15 weeks

Imaging comment

The imaging section is one of the strongest aspects of this paper. MRI demonstrated a **subacute intramural hematoma** within the left extracranial internal carotid artery associated with marked luminal stenosis but without cerebral infarction. Serial MRI documented the evolution of the hematoma from the subacute to the chronic phase together with normalization of the arterial lumen. CT angiography confirmed near-complete recanalization at follow-up. An additional strength of this report is the use of **tongue electromyography**, which initially showed acute denervation (fibrillation potentials and repetitive discharges) and normalized after recovery, providing objective evidence of reversible hypoglossal nerve dysfunction secondary to carotid compression.

Clinical comment

This case illustrates a classic presentation of **isolated hypoglossal nerve palsy secondary to spontaneous extracranial internal carotid artery dissection**, without Horner syndrome, stroke, or involvement of other cranial nerves. Unlike many previous reports, the diagnosis was supported not only by MRI and CT angiography but also by serial electromyography documenting denervation and subsequent reinnervation of the tongue. The complete clinical and radiological recovery after conservative antiplatelet therapy further supports the hypothesis that transient mechanical compression from the intramural hematoma was the principal mechanism of nerve dysfunction.

Ali et al.

Title: *Unilateral Hypoglossal Nerve Palsy: A Rare Presentation of Left Internal Carotid Artery Dissection*

Authors: Ahmed Ali, Ashok Mathews, Wedad Samy

Institution: North Bristol NHS Trust, Southmead Hospital, Bristol, United Kingdom

Study design: Case report

Table 4. Demographic and Etiopathogenetic Characteristics

Variable	Finding
Age	**76 years**
Sex	**Male**
Internal carotid artery dissection	**Yes**
Side of ICA dissection	**Left**
Aetiology	**Spontaneous**
Hypertension	**Yes**
Trauma	**No**
Minor cervical trigger	NR

Variable	Finding
Dental procedure	No
Chiropractic manipulation	No
Smoking	NR
Other vascular risk factors	NR

Table 5. Clinical and Imaging Characteristics

Variable	Finding
Isolated CN XII palsy	**Yes**
Other cranial nerve involvement	**No reported**
Collet–Sicard syndrome	**No**
Horner syndrome	**No reported**
Stroke / cerebral ischemia	**No**
Headache	NR
Neck pain	NR
Dysarthria	**Yes**
Dysphagia	**Yes**
Odynophagia	**Yes**
Tongue deviation	**Yes**
Tongue atrophy	NR
Tongue fasciculations	NR
MRI	**Yes**
CT	**Yes**
CTA	**Yes**
MRA	NR
Duplex ultrasound	NR
DSA	NR
Intramural hematoma	**Yes**
Luminal stenosis	NR
Double lumen	NR
Pseudoaneurysm	**No reported**

Variable	Finding
Direct CN XII compression demonstrated	**Probable (MRI findings)**

Table 6. Treatment and Outcome Characteristics

Variable	Finding
Conservative treatment	**Yes**
Antiplatelet therapy	**Aspirin**
Anticoagulation	No reported
Endovascular treatment	No
Surgical treatment	No
Clinical outcome	**Improvement**
Imaging follow-up	**CTA at 3 months**
Radiological outcome	**Healing/improvement of ICA dissection**
Follow-up duration	**3 months**

Imaging comment

The multimodal imaging approach included **CT, CTA, and fat-suppressed MRI**. CTA identified a **left internal carotid artery dissection at the skull base**, while fat-suppressed MRI confirmed the presence of an **intramural hematoma**, excluding acute ischemic stroke. Follow-up CTA performed three months later demonstrated **healing of the dissection**, with no residual focal narrowing or vascular irregularity. The imaging findings support the hypothesis of **mechanical compression of the hypoglossal nerve** by the mural hematoma.

Clinical comment

This case describes an **isolated unilateral hypoglossal nerve palsy** secondary to **left extracranial internal carotid artery dissection** in an elderly patient. Unlike the typical presentation of ICAD, the patient had **no ischemic manifestations**, and diagnosis relied on recognizing isolated lower cranial nerve dysfunction. The authors emphasize that carotid artery dissection should be considered in the differential diagnosis of isolated hypoglossal palsy, even in the absence of stroke, and that **CTA combined with fat-suppressed MRI** provides accurate diagnosis while conservative antiplatelet therapy is associated with a favorable clinical and radiological outcome.

Kordjian & Petersen, 2023

Title: *Hypoglossal nerve palsy in a patient with internal carotid dissection* (English Summary)

Original title: *N. hypoglossus-parese ved a. carotis interna-dissektion*

Authors: Hayarpi H. Kordjian, Anita G. Petersen

Journal: *Ugeskrift for Læger*

Year: 2023

Study design: Case report

Table 4. Demographic and Etiopathogenetic Characteristics

Variable	Finding
Age	**38 years**
Sex	**Male**
Internal carotid artery dissection	**Yes**
Side of ICA dissection	**Left**
Aetiology	**Spontaneous**
Trauma	**No major trauma**
Minor cervical trigger	**Previous chiropractic neck manipulation (2 weeks before symptom onset)**
Dental procedure	**Yes (root canal treatment 3 days before presentation)**
Hypertension	**Marked hypertension at presentation (190/130 mmHg), previously unknown**
Smoking	NR
Connective tissue disease	NR

Table 5. Clinical and Imaging Characteristics

Variable	Finding
Isolated CN XII palsy	**Initially yes**
Other cranial nerve involvement	**Glossopharyngeal nerve (CN IX) involvement developed during hospitalization**
Collet–Sicard syndrome	**No**
Horner syndrome	**No**
Stroke / cerebral ischemia	**No**
Headache	**Yes (severe)**
Neck pain	**Previous neck pain**
Mandibular pain	No
Dysarthria	**Yes**
Dysphagia	**Mild**
Tongue deviation	**Yes (left)**
Tongue swelling	**Apparent swelling of the tongue/root of tongue**
Tongue atrophy	**No**
MRI	**Yes**
MRA	**Yes**
CT brain	**Normal**

Variable	Finding
CTA	No
Duplex ultrasound	NR
DSA	No
Intramural hematoma	**NR**
Luminal stenosis	NR
Double lumen	NR
Pseudoaneurysm	**No**
ICA dissection location	**Approximately 3 cm distal to the carotid bifurcation**

Table 6. Treatment and Outcome Characteristics

Variable	Finding
Conservative treatment	**Yes**
Antiplatelet therapy	**Aspirin + Clopidogrel**
Anticoagulation	**No**
Endovascular treatment	**No**
Surgical treatment	**No**
Hospital stay	**3 days**
Clinical outcome	**Complete recovery**
Imaging follow-up	**MRI/MRA at 3 months**
Radiological outcome	**No residual abnormalities**
Follow-up duration	**3 months**

Imaging comment

Initial brain CT was normal, excluding acute intracranial pathology. Magnetic resonance angiography demonstrated a **left extracranial internal carotid artery dissection located approximately 3 cm distal to the carotid bifurcation**, establishing the diagnosis. Follow-up magnetic resonance imaging performed after three months showed **complete radiological resolution**, consistent with healing of the arterial dissection. Although detailed vascular features such as intramural hematoma morphology or luminal stenosis were not reported, the imaging findings were sufficient to confirm the diagnosis and monitor recovery.

Clinical comment

This report highlights the importance of considering **internal carotid artery dissection in patients presenting with isolated hypoglossal nerve palsy**, particularly when preceded by severe headache or recent cervical manipulation. A notable feature of this case is that the patient initially presented with **isolated CN XII palsy**, but subsequently developed **ipsilateral glossopharyngeal nerve involvement**, indicating progression from isolated cranial neuropathy to limited lower cranial nerve involvement. Conservative dual antiplatelet therapy resulted in complete clinical and radiological recovery within three months. The authors emphasize that early

recognition of carotid artery dissection is essential to prevent thromboembolic complications and that this diagnosis should be considered by neurologists, otolaryngologists, dentists, and emergency physicians.

REWIEW

Questa review di **Sturzenegger & Huber, 1993 non deve essere trattata come un caso clinico**, ma come una fonte secondaria che riassume tutta la letteratura disponibile fino al 1993.

Io farei una tabella separata (**Table 7**) dedicata esclusivamente alle review.

Table 7. Review articles on hypoglossal nerve palsy associated with internal carotid artery dissection

Study	Type	Patients included	Main findings	Relevance
Sturzenegger & Huber, 1993	Narrative review + 2 new cases	Review of 36 published cases + 2 original cases (38 total)	Hypoglossal nerve was the most frequently involved cranial nerve. Lower cranial nerve palsies are mainly attributed to compression by mural hematoma or pseudoaneurysm. MRI is emphasized as the most sensitive imaging modality for diagnosis.	Landmark review establishing the pathophysiology and imaging characteristics of cranial nerve palsy associated with ICA dissection.

Variable	Findings (38 patients)
Total patients	38
Original cases	2
Previously published cases	36
XII nerve involvement	27 (71%)
IX nerve involvement	18 (47%)
X nerve involvement	17 (45%)
XI nerve involvement	7 (18%)
Horner syndrome	20 (53%)
Cerebral ischemia	10 (26%)
MRI showing mural hematoma	

Table 7. Literature reviews on isolated hypoglossal nerve palsy associated with internal carotid artery dissection

Study	Study design	Cases reviewed	Main demographic findings	Main clinical findings	Imaging findings	Treatment	Main conclusions
**Mes et al., 2018**	Literature review + original case report	**22 published cases** *(original case excluded from pooled analysis)*	Predominantly males; most patients were **35–45 years** old; **2 bilateral ICADs** reported	Headache **60%**; neck pain **13.5%**; mandibular pain **9%**; dysarthria **68%**; dysphagia **52%**. Isolated CN XII palsy represented the only neurological deficit.	MRI/MRA identified intramural hematoma and ICA dissection; CTA was frequently used as complementary imaging. The predominant mechanism was **mechanical compression of CN XII by subadventitial intramural hematoma**.	**9 anticoagulation**, **5 antiplatelet therapy**; no evidence supporting superiority of either strategy (consistent with CADISS trial).	Largest review specifically focused on isolated hypoglossal nerve palsy caused by ICAD. Prognosis was generally favorable, with complete or substantial neurological recovery in most patients after conservative treatment.

.

Main findings extracted from the review

Variable	Findings
Published cases reviewed	**22**
New case presented	**1**
Total patients analyzed	**23**
Bilateral ICA dissections	**2 cases**
Most frequent symptom	Headache/neck pain
Hypoglossal palsy	**100%**
Horner syndrome	Rare
Stroke/cerebral ischemia	Rare
Most common imaging	MRI/MRA ± CTA
Main proposed mechanism	Direct compression by intramural hematoma
Outcome	Generally favorable with conservative treatment

Comment

Mes et al. performed the **first review exclusively dedicated to isolated hypoglossal nerve palsy secondary to internal carotid artery dissection**, identifying **22 published cases** and adding **one original patient**, for a total of **23 patients**. This work provides the most comprehensive synthesis currently available for this uncommon clinical presentation.

The review confirms that patients are typically **middle-aged males** presenting with **ipsilateral headache or neck pain**, followed by isolated tongue deviation due to XII cranial nerve palsy. Unlike classical ICAD, **Horner syndrome and cerebral ischemia are uncommon**, emphasizing that local compressive symptoms may represent the only clinical manifestation.

From a radiological perspective, the authors show that **MRI combined with MRA**, often complemented by **CTA**, represents the most reliable diagnostic strategy because it allows direct visualization of the **intramural hematoma**, the degree of luminal narrowing, and the close anatomical relationship between the dissected artery and the hypoglossal nerve.

Regarding pathophysiology, the review strongly supports the **compressive hypothesis**, whereby a **subadventitial intramural hematoma** expands externally into the parapharyngeal space, compressing the hypoglossal nerve. The alternative mechanism of ischemic injury to the vasa nervorum is acknowledged but considered less strongly supported by the available imaging findings.

Finally, the pooled cases indicate an overall **favorable prognosis**, with most patients experiencing complete or substantial neurological recovery following conservative medical therapy (antiplatelet or anticoagulant treatment), whereas invasive procedures were required only exceptionally.

Cruciata et al., 2017

Title: Hypoglossal nerve palsy caused by spontaneous internal carotid artery dissection

Authors: Cruciata et al.

Journal: Neurological Sciences

Year: 2017

Study design: Case report

Table 4. Demographic and Etiopathogenetic Characteristics

Variable	Finding
Age / Sex	56 years / Male
Smoking	NR
Hypertension	Yes
Connective tissue disorder / genetic disease	No
Other vascular risk factors	None reported
Trauma	No
Minor cervical mechanical trigger	No
Recent infection	No
Dental procedure	No
Chiropractic manipulation	No
Vigorous cough / Valsalva	No
Other predisposing factor	Spontaneous ICAD

Table 5. Clinical and Imaging Characteristics

Variable	Finding
Internal carotid artery dissection	Yes (Left ICA)
Headache	Yes

Variable	Finding
Neck pain	Yes
Dysarthria	Yes
Dysphagia	No
Tongue deviation	Yes
Tongue weakness	Yes
Tongue atrophy	No
Isolated CN XII palsy	Yes
Other cranial nerve involvement	No
Horner syndrome	No
Stroke / cerebral ischemia	No
MRI	Yes
MRA	Yes
CTA	No
CT	Yes
Doppler ultrasound	No
Duplex ultrasound	No
DSA	No
Intramural hematoma	Yes
Luminal stenosis	Yes
ICA occlusion	No
External arterial enlargement	Yes
Pseudoaneurysm	No
Direct CN XII compression demonstrated	No
Mass effect on CN XII	Suggested

Table 6. Treatment and Outcome Characteristics

Variable	Finding
Conservative treatment	Yes
Antiplatelet therapy	Yes
Anticoagulation	No
Endovascular treatment	No

Variable	Finding
Clinical outcome	Complete recovery
Imaging follow-up	Yes
Persistent vascular abnormality	No
Recurrence	No

Imaging comment

MRI demonstrated the typical appearance of extracranial ICA dissection with intramural hematoma and mural expansion without evidence of cerebral ischemia. Although direct compression of the hypoglossal nerve was not demonstrated, the imaging findings supported a compressive mechanism.

Table 1

aggiunge

Male

56 years

Hypertension

Headache

Neck pain

Dysarthria

Isolated CN XII

No Horner

No stroke

Table 2

aggiunge

MRI

MRA

CT

Intramural hematoma

Luminal stenosis

External arterial enlargement

Suggested mass effect

No direct compression demonstrated

Table 3

aggiunge

Antiplatelet therapy

Conservative treatment

Complete recovery

Imaging follow-up

No recurrence

De Santis et al., 2012

Title: Internal carotid artery dissection presenting as isolated hypoglossal nerve palsy

Authors: De Santis et al.

Journal: Neurological Sciences

Year: 2012

Study design: Case report

Table 4. Demographic and Etiopathogenetic Characteristics

Variable	Finding
Age / Sex	Female
Smoking	NR
Hypertension	No
Connective tissue disorder	No
Other vascular risk factors	None reported
Trauma	No
Minor cervical mechanical trigger	No
Recent infection	Yes
Dental procedure	Yes
Chiropractic manipulation	No
Vigorous cough / Valsalva	No
Other predisposing factor	None reported

Table 5. Clinical and Imaging Characteristics

Variable	Finding
Internal carotid artery dissection	Yes (Left ICA)
MRI	Yes
MRA	Yes
CTA	No
CT	Yes
Doppler ultrasound	Yes
Duplex ultrasound	No
DSA	No

Variable	Finding
Intramural hematoma	Yes
Luminal stenosis	Yes
External arterial enlargement	Yes
Pseudoaneurysm	No
Direct CN XII compression demonstrated	No
Mass effect on CN XII	Yes
Stroke / cerebral ischemia	No

Table 6. Treatment and Outcome Characteristics

Variable	Finding
Conservative treatment	Yes
Antiplatelet therapy	Yes
Clinical outcome	Complete recovery
Imaging follow-up	Ultrasound
Persistent vascular abnormality	No
Recurrence	No

Imaging comment

MRI demonstrated intramural hematoma with mural expansion causing mass effect at the skull base. Ultrasound follow-up documented vascular improvement without evidence of pseudoaneurysm.

Table 1

aggiunge

Female

Recent infection

Dental procedure

Isolated CN XII

No stroke

Table 2

aggiunge

MRI

MRA

CT

Doppler ultrasound

Intramural hematoma

Luminal stenosis

External arterial enlargement

Mass effect

No pseudoaneurysm

Table 3

aggiunge

Antiplatelet therapy

Conservative treatment

Complete recovery

Ultrasound follow-up

Qi et al., 2015

Table 1

aggiunge

Female

45 years

Isolated CN XII

No vascular risk factors

Table 2

aggiunge

MRI

MRA

CTA

Intramural hematoma

Luminal stenosis

External arterial enlargement

Suggested mass effect

Table 3

aggiunge

Anticoagulation

Complete recovery

Imaging follow-up

Pongmoragot et al., 2013

Table 1

aggiunge

Male

60 years

Hypertension

Minor cervical mechanical trigger (heavy lifting)

Dysarthria

Isolated CN XII

Table 2

aggiunge

MRI

MRA

CTA

Intramural hematoma

Luminal stenosis

External arterial enlargement

Mass effect

Table 3

aggiunge

Antiplatelet therapy

Conservative treatment

Complete recovery

Mathey et al., 2010

Table 1

aggiunge

Male

56 years

Spontaneous ICAD

Isolated CN XII

Table 2

aggiunge

MRI

MRA

Intramural hematoma

Luminal stenosis

External arterial enlargement

No pseudoaneurysm

Table 3

aggiunge

Anticoagulation

Complete recovery

Imaging follow-up
