# Supplementary material for: Isolated Hypoglossal Nerve Palsy Associated with Internal Carotid Artery Dissection: A Systematic Review
Source: Neurol Int. 2026 Aug 14;18(8):150. doi: 10.3390/neurolint18080150 (PMC13517042; doi:10.3390/neurolint18080150)
Supplement: Supplementary file 1 [file neurolint-18-00150-s001.zip › Supplementary Table S3A.pdf]

Table S3A: JBI critical appraisal of the included case reports

**Authors**	**Study Type**	**Instrument**	**Score**
Ursekar MA, Singhal BS, Konin BL	Case report	JBI Case Report	**8/8**
M. Boukobza 1993	Case report	BI Case Report	8/8
Shahab R, Savy LE, Croft CB, Hung T	Case report	JBI Case Report	**8/8**
Lindsay FW, Mullin D, Keefe MA	Case report	JBI Case Report	**8/8**
Hafkamp HC, van der Goten A, Manni JJ	Case report	JBI Case Report	**8/8**
Freilinger TM, Heuck A, Strupp M, Jund R	Case report	JBI Case Report	**8/8**
Spitzer C, Mull M, Töpper R	Case report	JBI Case Report	**8/8**
Fujii H, Ohtsuki T, Takeda I, Hosomi N, Matsumoto M	Case report	JBI Case Report	**8/8**
Riancho J, Infante J, Mateo JI, Berciano J, Agea L	Case report	JBI Case Report	**8/8**
Ferlazzo E, Gasparini S, Arcudi L, Versace P, Aguglia U	Case report	JBI Case Report	**7/8**
Joshi P, Bourke D	Case report	JBI Case Report	**7/8**
Allingham W, Devakumar V, Herwadkar A, Punter M	Case report	JBI Case Report	**7/8**
Okunomiya T, Kageyama T, Suenaga T	Case report	JBI Case Report	**7/8**
Chen Z, Yuan J, Li H, et al.	Case report	JBI Case Report	**8/8**
Abukeshek T, Gbande P, Hamed R	Case report	JBI Case Report	**8/8**
Ali A, Mathews A, Samy W	Case report	JBI Case Report	7/8
Marin et al.	Case report	JBI Case Report	6/8
Kordjian HH, Petersen AG. (2023)	Case report	JBI Case Report	7/8
Mathey DG et al. (2010)	Case report	JBI Case Report	**6/8**
Pongmoragot J et al. (2013)	Case report	JBI Case Report	**8/8**
Qi B et al. (2015)	Case report	JBI Case Report	**7/8**
De Santis F. et al. (2012)	Case report	JBI Case Report	**8/8**
Cruciata G. et al (2017)	Case report	JBI Case Report	**8/8**
