# Supplementary material for: Isolated Hypoglossal Nerve Palsy Associated with Internal Carotid Artery Dissection: A Systematic Review
Source: Neurol Int. 2026 Aug 14;18(8):150. doi: 10.3390/neurolint18080150 (PMC13517042; doi:10.3390/neurolint18080150)
Supplement: Supplementary file 1 [file neurolint-18-00150-s001.zip › Supplementary Table S3B.pdf]

Supplementary Table S3B

Supplementary Table S3B: JBI critical appraisal of the included case series

Study	JBI critical appraisal tool	Score
Sturzenegger and Huber, 1993	JBI Critical Appraisal Checklist for Case Series	5/10
Olzowy et al., 2006	JBI Critical Appraisal Checklist for Case Series	5/10
Torbus-Paluszczak et al., 2018	JBI Critical Appraisal Checklist for Case Series	5/10
Jurkiewicz et al., 2019	JBI Critical Appraisal Checklist for Case Series	4/10

Note:Scores were calculated by assigning one point to each item rated "Yes"; items rated "No", "Unclear", or "Not Applicable" received no points. The maximum possible score was 10. Sturzenegger and Huber reported two original cases together with a narrative literature review; the original case-series component was appraised in this table, whereas its narrative component was assessed separately in Supplementary Table S3C. Torbus-Paluszczak et al. reported two patients, only one of whom fulfilled the eligibility criteria of the present review; nevertheless, the publication was classified and appraised according to its original case-series design.
