# Supplementary material for: Isolated Hypoglossal Nerve Palsy Associated with Internal Carotid Artery Dissection: A Systematic Review
Source: Neurol Int. 2026 Aug 14;18(8):150. doi: 10.3390/neurolint18080150 (PMC13517042; doi:10.3390/neurolint18080150)
Supplement: Supplementary file 1 [file neurolint-18-00150-s001.zip › Supplementary Table S3C.pdf]

Supplementary Table S3C

Supplementary Table S3C: JBI critical appraisal of the narrative review components.

Publication	JBI critical appraisal tool	Score
Sturzenegger and Huber, 1993	JBI Critical Appraisal Checklist for Textual Evidence: Narrative	6/6
Mes et al., 2018	JBI Critical Appraisal Checklist for Textual Evidence: Narrative	6/6

Note: Scores were calculated by assigning one point to each item rated "Yes"; items rated "No", "Unclear", or "Not Applicable" received no points. The maximum possible score was 6. The appraisal reported in this table refers exclusively to the narrative review components of the two hybrid publications. Their original eligible patient data were methodologically appraised separately according to the design of the original case-report or case-series component.
